# Supplementary material for: ArcA positively regulates the expression of virulence genes and contributes to virulence of porcine Shiga toxin-producing enterotoxigenic Escherichia coli
Source: Microbiol Spectr. 2023 Nov 2;11(6):e01525-23. doi: 10.1128/spectrum.01525-23 (PMC10714933; doi:10.1128/spectrum.01525-23)
Supplement: Supplemental material — Fig. S1 to S4; Tables S1 and S2. [file spectrum.01525-23-s0001.pdf]

ArcA positively regulates the expression of virulence genes and contributes to virulence of porcine Shiga toxin-producing enterotoxigenic *Escherichia coli*

Running title: ArcA role in enterotoxigenic *E. coli* virulence

Fengwei Jiang[a,b], Yan Yang[a], Zhao Mao[a], Wentong Cai[a,#], Ganwu Li[a,b,#]

[a] State Key Laboratory for Animal Disease Control and Prevention, Harbin Veterinary Research Institute, Chinese Academy of Agricultural Sciences, Harbin, China; [b]Department of Veterinary Diagnostic and Production Animal Medicine, College of Veterinary Medicine, Iowa State University, Ames, Iowa, USA;

[#]Address correspondence to: Ganwu Li, liganwu@iastate.edu; Wentong Cai, caiwentong@caas.cn

Tel: +86- 451-51997177

Fax: +86- 451-51997166

Abstract

Enterotoxigenic *Escherichia coli* (ETEC) cause severe diarrhea in humans and animals. These bacteria encode two major classes of virulence factors, adhesins that promote colonization of the small intestine and enterotoxins that induce fluid secretion and thus diarrhea. Oxygen and redox sensors are regulators of virulence expression in multiple bacterial pathogens. In this study, we show that microaerobic conditions enhance the expression of virulence factors in a porcine Shiga toxin-producing ETEC (STEC/ETEC) strain. We then investigated role of the aerobic respiration control regulator ArcA in the pathogenicity and virulence regulation in STEC/ETEC. In a mouse model, deletion of *arcA* caused less severe pathology, and the *arcA* mutant displayed lower levels of colonization and obvious weight gain of the mice compared to the wild type. In a cell culture model, a lack of *arcA* reduced the adherence of STEC/ETEC to porcine intestinal epithelial cells. Furthermore, ArcA positively regulated the expression of several key virulence factors, including F18 fimbriae (*fed*), heat-labile (*eltA*) and heat-stable (*estB*) toxins, Shiga toxin 2e (*stx2e*), and hemolysin (*hlyC*), under microaerobic conditions and *in vivo conditions*. We then found that ArcA positive regulated the expression of *eltA*, *estB*, and *hlyC* by competing with a global repressor H-NS. Mechanistically, we show that ArcA protein directly binds to the promoters of target genes, displaces H-NS silencing from the promoter and counteracts H-NS's repression. Collectively, our data established a key role for ArcA in the pathogenicity and virulence expression of porcine STEC/ETEC.

Importance

Enterotoxigenic *Escherichia coli* (ETEC) cause severe diarrhea in humans and animals, leading to death and huge economic loss worldwide. Thus, elucidation of ETEC's pathogenic mechanisms will provide powerful data for the discovery of drugs serving as prevention or therapeutics against ETEC caused diarrheal diseases. Here, we report that ArcA plays an essential role in the pathogenicity and virulence regulation in ETEC by positively regulates the expression of several key virulence factors including F18 fimbriaea, heat-labile and heat stable toxins, shiga toxin 2e, and hemolysin, under microaerobic conditions and *in vivo.* Moreover, we found that positive regulation of several virulence genes by ArcA requires a global repressor H-NS, implying that ArcA may exert positive effects by antagonizing H-NS. Collectively, our data established a key role for ArcA in the pathogenicity of porcine ETEC and ETEC strains isolated from human infections. Moreover, our work reveals another layer of regulation in relation to oxygen control of virulence factors in ETEC.

Keywords: Shiga toxin-producing enterotoxigenic *Escherichia coli* (STEC/ETEC), ArcA, virulence regulation, attenuation, virulence factor

Introduction

Enterotoxigenic *Escherichia coli* (ETEC) is a prominent enteric pathogen responsible for causing tens of millions of cases of diarrheal illnesses annually. Its impact on young children, particularly those under the age of 5, is of significant concern due to their heightened vulnerability to ETEC infection, especially in regions where the pathogen is endemic. In 2015, ETEC was responsible for an estimated 100 million cases of diarrhea, a staggering figure that underscores the severity of the issue, tragically resulting in approximately 60,000 fatalities within this vulnerable age group (1, 2). ETEC is also a very common cause of diarrhea in agricultural production animals, including pigs, cattle, and sheep (3, 4). Diarrhea caused by ETEC in neonatal and post-weaning pigs is an economically significant disease worldwide due to mortality, weight loss, slow growth, and treatment cost (5). ETEC enter animals via the oral route usually in the form of contaminated food or water and after passing through the stomach, these bacteria can attach to the intestinal epithelium using fimbrial or non-fimbrial adhesins. Adhesins frequently carried by animal/human ETEC include K88 (also called F4), F18, K99 (F5), 987P (F6) and F41. Once colonized, ETEC can multiply rapidly and secrete one or more classes of enterotoxins, such as heat-labile enterotoxin (LT) and heat-stable enterotoxin (ST). These toxins can induce the loss of water and electrolytes from epithelial cells into the gut lumen, and subsequently the onset of diarrhea (4, 6). Some animal/human ETEC strains carry Shiga toxin (Stx) type 2e, giving rise to hybrid Shiga toxin-producing enterotoxigenic *E. coli* (STEC/ETEC) (7, 8). Stx2 variant “e” encoded by the *stx2e*AB operon is the

most frequent *stx2* variant found in porcine fecal samples (9-11) and is highly associated with severe edema disease in pigs, which is also called edemigenic toxin (12). STEC/ETEC can induce both diarrhea and edema, leading to lethality in pigs (13, 14). Therefore, understanding its pathogenesis is of great significance and may aid in the development of therapeutics and antimicrobials.

Among all the adhesins, F18 is highly associated with strains causing porcine edema and post-weaning diarrhea (PWD) (10, 15, 16). F18 fimbriae is encoded by the *fed* (fimbriae associated with edema disease) gene locus comprised of 6 genes. The *fedA* gene encodes the major subunit of the fimbriae, *fedB* the usher protein, *fedC* the periplasmatic chaperone, *fedE* a small linker protein between FedA and FedF, and *fedF* the fimbrial adhesin (17, 18). FedF acts as an adhesin specific for porcine intestinal epithelial cells, and FedF is highly conserved in both F18ab and F18ac serological subtypes (19). In situations where the host is exposed to ETEC, and inhibitory milk glycans are absent after weaning, FedF has the capability to bind to a range of hexoses present in the glycosphingolipids of the intestinal cell membrane. This binding ability facilitates the initial attachment process (20).

Upon attachment, ETEC strains express one or both of the two classical toxins, LT and ST. Encoded by the *eltAB* operon, LT is a heterohexameric protein of ~84 kDa containing one A subunit (LTA) and five identical B subunits (LTB) (21). ST toxins are a group of low-molecular-weight enterotoxigenic peptides, usually containing 18 or 48 amino acids. STs are divided into two categories: the human (STh) and porcine (STp) ST based on their preferential hosts (22); or type 1 (ST1 or STa) and type 2

(ST2 or STb) ST based on their pathogenicity and solubility in methanol (23-25). STa and STb are encoded by *estA* and *estB,* respectively. Upon binding to their receptors, LT and ST trigger a cascade of cellular pathways, eventually leading to the transport of Cl^- and HCO_3^- to the intestinal lumen, as well as the loss of water (22, 26). ETEC strains may also possess hemolytic toxins, such as hemolysin (encoded by the *hlyCABD* operon) and cytolysin (encoded by *clyA* or *hlyE*) (27, 28). Although an ETEC strain containing only one adhesin and one enterotoxin is adequate to cause symptomatic diseases, hemolytic ETEC strains display increased virulence (29).

Previous studies demonstrate that the expression of some ETEC adhesins and enterotoxins are modulated in response to host environments, including pH, bile, and osmolarity (30). Two regulatory proteins CRP and H-NS play a key role in integrating extracellular signals such osmolarity and metabolism into the control of toxin production (31). A recent study shows that oxygen tension in the intestinal environment also influences the expression of genes encoding LT and the CFA/I adhesin. In particular, under aerobic conditions, there was a substantial increase in the expression of LT and the CFA/I adhesion, in stark contrast to the anaerobic conditions. Intriguingly, there was no significant difference between the expression of these factors under aerobic and microaerobic conditions. However, it is worth noting that the expression of ST2 (heat-stable toxin 2) exhibited a significant increase under microaerobic conditions when compared to aerobic conditions. The global regulator FNR (*f*umarate and *n*itrate *r*eduction) represses the expression of ETEC virulence factors (VFs) in the anaerobic lumen, and this repression can be relieved when ETEC

nears the intestinal epithelium, where leaked oxygen from the epithelial cells inactivates FNR (32).

Similar to FNR, the Arc two-component signal transduction system, comprised of the kinase sensor ArcB and its cognate response regulator ArcA, enables *E. coli* adaptation to changing oxygen availability (33). ArcB kinase activity is modulated by the redox state of the ubiquinone and menaquinone pools (34). In response to low or no availability of oxygen, ArcB phosphorylates and activates ArcA, which then inhibits the expression of genes required for aerobic metabolism but stimulates genes implicated in the anaerobic respiratory and metabolic pathways (35, 36). Although the ArcA regulon has been extensively studied in *E. coli* K-12 strain (35-37), the role of ArcA for virulence and virulence regulation is less well studied in pathogenic *E. coli*. In this study, using a porcine hybrid STEC/ETEC strain (MD724-020) isolated from a piglet with acute diarrhea, we show that microaerobic conditions enhance the expression of VFs relative to anaerobic conditions and that ArcA positively regulates the expression of enterotoxins and hemolysin under microaerobic conditions, contributing substantially to the virulence of ETEC. Therefore, this work reveals another layer of regulation in relation to oxygen control of VFs in ETEC.

Results

Microaerobic conditions enhance the expression of genes encoding enterotoxins and hemolysin

While the intestinal lumen is commonly thought to be anaerobic, the tissue surface surrounding the lumen can be microaerobic due to oxygen leaked from intestinal

epithelial cells. Although accurate data about oxygen concentrations in the porcine intestine are lacking, noninvasive imaging of oxygen levels in the gastrointestinal tract of living mice indicated that the milieu in the mid-small intestine is microaerobic (1.4% of atmospheric pressure) (38). To assess whether the expression of genes encoding adhesin and toxin is different between microaerobic and anaerobic conditions, RT–qPCR assays were carried out with six selected genes in the porcine hybrid STEC/ETEC strain MD724-020 under both microaerobic (6-12% O_2) and anaerobic (21% CO_2) conditions, which mimic *in vivo* conditions. As shown in Fig. 1, the expression of *eltA*, *estB, stx2e, hlyA* and *hlyE* was significantly higher under microaerobic conditions than under anaerobic conditions, while the expression of the adhesin gene *fedA* was undetectable under both microaerobic and anaerobic conditions (data not shown). The largest fold changes were observed in *stx2e* and *hlyC,* with a >12-fold change and a > 5-fold change, respectively. Given that the milieu environment in the mid-small intestine is likely microaerobic and the expression of several major virulence genes of porcine STEC/ETEC was higher in microaerobiosis than in anaerobiosis, microaerobic conditions were used for all expression assays and comparisons throughout this study.

Deletion of *arcA* significantly attenuates STEC/ETEC virulence both *in vivo* and *in vitro*

To address the role of ArcA in STEC/ETEC, the virulence of the wild-type MD724-020 Δ*arcA* mutant and Δ*arcA* complementation strains was compared using a 4-week-old mouse model. We first demonstrate that WT, Δ*arcA* mutant and Δ*arcA*

complementation strains grew at similar rates under aerobic, microaerobic or anaerobic conditions (Fig. S1). Mice were pretreated with an antibiotic cocktail prior to infection. The WT, Δ*arcA* mutant and Δ*arcA* complementation strains were inoculated into eight mice (n = 8/group) with an inoculation dose of 1×10^9 CFU. We observed that three mice in the WT and two in the Δ*arcA* complemented strain challenged group died within 15 days post-infection (dpi). The survivors displayed signs of distress and exhibited bloody stool. In stark contrast, in the group infected with the Δ*arcA* mutant, there were no recorded deaths or apparent symptoms at 15 dpi mark. Although the difference between the WT and Δ*arcA* mutant groups is not statistically significant (P = 0.0888, Fig. 2A), there is a trend that loss of *arcA* can increase the survival of mice. Additionally, the body weights of the surviving mice were monitored for two weeks. As shown in Fig. 2B, the WT and Δ*arcA* complementation strain-challenged mice showed significant weight losses from 2 to 15 dpi compared with the Δ*arcA* mutant-challenged mice ($P < 0.01$). The CFU analysis of fecal samples confirmed the absence of the STEC/ETEC strains before challenge. Fecal shedding of STEC/ETEC in each group was calculated by determining the CFUs of STEC/ETEC on blood agar, and no significant differences in shedding were observed between the Δ*arcA* and WT groups at 1 dpi. As shown in Fig. 2C, Δ*arcA* strain shedding was significantly lower than WT at two and three dpi ($P <$ 0.01 and $P < 0.05$). Three days later, fecal shedding of most mice dropped to undetectable level. Besides, histopathological examination detected pronounced changes in the WT and Δ*arcA* complementation strain-challenged mice but not in the

mice challenged by the Δ*arcA* mutant. WT and Δ*arcA* complementation strain infection caused atrophy of microvilli and a large number of inflamed cells in the lamina propria of the ileum, but nearly no histological changes were observed in the mice infected with the Δ*arcA* mutant (Fig. 2D). These results demonstrate that the Δ*arcA* mutation significantly attenuates STEC/ETEC virulence in the murine model.

To determine whether *arcA* affects the adherence of STEC/ETEC to porcine intestinal enterocytes isolated from jejunum, cultured IPEC-J2 cells were infected with the WT and Δ*arcA* mutant strains. As shown in Fig. 2E, deletion of *arcA* reduced STEC/ETEC adherence to IPEC-J2 cells by sixfold compared with that of the WT strains ($P < 0.01$). Complementation of the Δ*arcA* mutant by reintroduction of *arcA* greatly increased the levels of adherence. Therefore, these results suggest that *arcA* plays an important role in the adherence of STEC/ETEC to host cells.

ArcA regulates the expression of *fedABCDEF* encoding F18 pilus

The F18 pilus encoded by the *fed* gene cluster *fedABCDEF* mediates colonization of both ETEC and Shiga-toxin producing *E. coli* (STEC) in weaned pigs (39). We deleted the gene cluster *fedABCDEF* from the WT, and the adherence assay showed that a lack of the *fedABCDEF* operon dramatically reduced STEC/ETEC adherence to IPEC cells (Fig. 3A) ($P < 0.01$). To determine whether and how ArcA regulates the expression of *fedABCDEF*, we first used a reverse transcription-PCR assay to show that the gene cluster *fedABCDEF* forms one operon (Fig. 3B). The transcription start site (TSS) of the *fedABCDEF* operon was then mapped to the position 191 bp upstream of the *fedA*-coding region by using 5' RACE PCR (Fig. S2), and putative

-10 and -35 motifs of the promoter were identified as shown in Fig. 3C. Notably, when gene transcription levels were studied using chromosomal *fedA-lacZ* fusion and RT-qPCR under laboratory conditions in TSB and LB media, *fedABCDEF* expression was not detectable in the WT, Δ*arcA* mutant or Δ*arcA* complementation strain. The hemagglutination assay results also revealed that both the wild-type strain and the ΔarcA complementation strains possess the capability to agglutinate chicken red blood cells, leading to their clumping together. In contrast, the ΔarcA mutant strain was found to be incapable of achieving this agglutination, as shown in Figure 3D. During interaction with IPEC-J2 cells and infection in mice, the expression of *fedABCDEF* was detectable in the WT and Δ*arcA* complementation strains, but not in the Δ*arcA* mutant (Fig. 3E and 3F), suggesting a positive role of ArcA in the expression of *fedABCDEF* during ETEC interaction with IPEC-J2 cells or during infection *in vivo*.The potential binding site of ArcA was identified with the Prodoric Virtual Footprint program (version 3.0) (40) at positions +15 to +24 downstream of the TSS of *fedABCDEF* (Fig. 3C). The ArcA-His_6 fusion protein was able to shift the DNA fragments containing the *fedA* promoter region but not the control fragment within the coding region (Fig. 3G). Altogether, these data suggest that ArcA likely regulates the expression of the *fedABCDEF* operon in a direct manner.

Deletion of *arcA* significantly decreases the expression of heat-labile enterotoxin A *in vitro* and *in vivo*

The heat-labile enterotoxin A encoded by *eltA* is one of the well-known VFs for ETEC. To investigate whether and how ArcA regulates *eltA* expression, the TSS of

eltA was mapped to the position 54 bp upstream of the *eltA*-coding region by 5' RACE (Fig. S2), and the corresponding -10 and -35 motifs were identified at the proper distance to the TSS (Fig. 4A). The transcription of *eltA* was studied using chromosomal *eltA-lacZ* reporter gene fusion. Under microaerobic conditions, the expression of *eltA* was significantly reduced in the Δ*arcA* mutant in comparison to the WT ($P < 0.01$). Reintroduction of a plasmid carrying *arcA* restored *eltA-lacZ* expression at the transcriptional level (Fig. 4B). The expression of the EltA heat-labile toxin (EltA expression) in both the Δ*arcA* mutant and WT strain was assessed through western blot analysis, employing a monoclonal antibody specific to EltA. As illustrated in Figure 4C, the production of the EltA toxin exhibited a marked reduction in the Δ*arcA* mutant when compared to the WT strain, and this production was restored in the ΔarcA complemented strain (Grayscale analysis was conducted using ImageJ for quantification). In addition, the expression of *eltA* during ETEC adherence to IPEC-J2 cells and during infection in mice was also compared in WT, Δ*arcA* and Δ*arcA* complementation strains by RT–qPCR. As shown in Fig. 4D and 4E, the expression of *eltA* was significantly reduced in the Δ*arcA* mutant in comparison to that in the WT, and the expression levels increased in the Δ*arcA* complementation strain. These results indicate that ArcA regulates the expression of *eltA* under *in vitro* and *in vivo* conditions.

The potential binding sites of ArcA were identified by the Prodoric Virtual Footprint program (40). EMSAs showed that the ArcA protein shifted a DNA probe containing the potential ArcA binding sites but not the control DNA probe containing the coding

region of *eltA* (Fig. 4F). Together, these data demonstrate that ArcA directly interacts with the *eltA* promoter and affects the expression of heat-labile enterotoxin gene A.

Deletion of *arcA* significantly decreases the expression of heat-stable toxin B *in vitro* and *in vivo*

The heat-stable toxin (ST) is another major enterotoxin produced by ETEC (24, 25), and the *estB* gene encodes STp (23). To investigate whether and how ArcA regulates *estB* expression, the TSS was identified by 5' RACE PCR (Fig. 5A and S2), and the expression of *estB* was studied using chromosomal *estB-lacZ* reporter gene fusion. As shown in Fig. 5B, the expression of *estB* in the Δ*arcA* mutant was 2.6-fold lower than that in the WT under microaerobic conditions ($P < 0.01$). The production of STp heat-stable toxin (EstB expression) in the Δ*arcA* mutant and WT strain was further determined by ELISA using a monoclonal antibody against STp. As shown in Fig. 5C, the production of STp toxin was significantly lower in the Δ*arcA* mutant compared with the WT, and the production was restored in the Δ*arcA* complemented strain. Furthermore, the expression of *estB* was significantly lower in the Δ*arcA* mutant compared with that in the WT ($P < 0.01$) during ETEC adhering to intestinal porcine enterocytes (Fig. 5D) and during infection in the mouse intestine (Fig. 5E). These results demonstrate that ArcA positively regulates the expression of heat-stable toxin B *in vitro* and *in vivo*.

The ArcA binding site in the promoter region of the *estB* gene was predicted (Fig. 5A), and the DNA fragments containing the potential binding sites were amplified by PCR. EMSAs showed that the ArcA protein shifted the DNA fragment containing the

potential ArcA binding sites but not the negative control DNA fragments (Fig. 5F). These results suggest that ArcA could directly regulate the expression of the heat-stable toxin.

ArcA upregulates *stx2e* under *in vitro* and *in vivo* conditions

The ETEC strain used in this study possesses a *stx2e* gene encoding the edema toxin. The transcription start site of *stx2e* was determined by 5' RACE PCR (Fig. S2), and the -35 and -10 motifs of the promoter were identified (Fig. 6A). The expression of *stx2e* was studied using chromosomal *stx2e-lacZ* reporter gene fusion. As shown in Fig. 6B, the expression of *stx2e* in the Δ*arcA* mutant was 3-fold lower than that in the WT under microaerobic conditions ($P < 0.05$). The regulation of Stx2e by ArcA was additionally validated through Western blot analysis. As illustrated in Figure 6C, the Stx2e production in the Δ*arcA* mutant exhibited a notable and statistically significant decrease when compared to that in the WT (as depicted in Fig. 6C), with a p-value less than 0.01. The transcription of *stx2e* was also investigated in WT, Δ*arcA* and Δ*arcA* complemented strains during ETEC adhering to intestinal porcine enterocytes (Fig. 6D) and during infection in the mouse intestine (Fig. 6E). Our results showed that *stx2e* was expressed at much higher levels in the WT and the complemented strains than in the Δ*arcA* mutant. These results indicate that ArcA positively affected the expression of *stx2e*. EMSAs further showed that the ArcA protein could bind to the promoter region of the *stx2e* gene (Fig. 6F), suggesting that ArcA may directly regulate the expression of *stx2e*.

ArcA regulates the expression of hemolysin encoded by *hlyA*

Many ETEC strains encoding *hlyCABD* and/or *hlyE* are hemolytic (41, 42). For the STEC/ETEC strain used in this study, deletion of *hlyA*, but not *hlyE*, completely abolished the hemolytic activity, suggesting that *hlyA*, but not *hlyE*, is responsible for the hemolytic phenotype in the MD724-020 strain (Fig. 7A). To investigate whether ArcA regulates the expression of *hlyCABD*, the TSS of *hlyCABD* was obtained by 5' RACE PCR (Fig. 7B and S2), and the transcription of the *hlyCABD* operon was studied using chromosomal *hlyC-lacZ* reporter gene fusion. As shown in Fig. 7C, the expression level of *hlyC* was significantly lower in the Δ*arcA* mutant compared with that in the WT ($P < 0.01$) when cultured in TSB under microaerobic conditions. The regulation of *hlyCABD* by ArcA was further confirmed by western blot. As shown in Fig. 7D, the production of HlyA in the Δ*arcA* mutant was decreased by ~4-fold compared with that in the WT (grayscale as determined by ImageJ). Similarly, the expression of *hlyC* was significantly lower in the Δ*arcA* mutant compared with that in the WT during ETEC adhering to intestinal porcine enterocytes (Fig. 7E) and during infection in the mouse intestine (Fig. 7F) ($P < 0.01$). Furthermore, EMSAs were performed to test whether the ArcA regulator protein can bind to the promoter region of *hlyCABD*. As shown in Fig. 7G, the ArcA protein was able to shift DNA fragments containing the *hlyCABD* promoter region but not the negative control probe. Taken together, these results suggest that ArcA regulates the expression of the *hlyCABD* operon likely in a direct fashion, thus contributing to the hemolytic activity of ETEC.

ArcA positively regulates *eltA*, *estB* and *hlyC* by counteracting H-NS's repression

Many activators upregulate gene expression by counteracting H-NS's repression (43,

44). Previous reports have suggested that ArcA's positive regulation of target genes may involve H-NS (45, 46). Using two single mutants, Δ*hns* and Δ*arcA,* as well as a double mutant, Δ*hns*Δ*arcA*, we found that ArcA activates the expression of virulence genes (*eltA*, *estB* and *hlyC*) but H-NS represses their expression, and that the transcription of these genes is comparable between Δ*hns* and Δ*hns*Δ*arcA* (Fig. 8A to 8C), suggesting that positive regulation of these virulence genes by ArcA requires H-NS. Notably, regulation of *stx2e* by ArcA did not require H-NS, since the expression of *stx2e* was detectable in the WT (CT value 23.33±0.85 of *stx2e* versus 22.39±0.45 of *tus*), but not detectable in Δ*arcA*, Δ*hns* and Δ*hns*Δ*arcA* strains by RT-qPCR, suggesting a different regulatory mechanism. We then hypothesize that ArcA is able to displace H-NS from DNA *in vitro*, thereby relieving its repression. We first used EMSA to show that H-NS directly binds to the promoter regions of ArcA-regulated *eltA* (Fig. 8D). To examine competition between ArcA and H-NS, we set up reactions with both H-NS and ArcA added to the *eltA* promoter probe. These reactions contain a constant amount of H-NS but varying concentrations of ArcA. We observed that increasing concentrations of ArcA incrementally shifted the migration of the DNA-protein complex to resemble that of the ArcA-*eltA* complex (Fig. 8E). This result suggests that ArcA outcompete H-NS in binding to the promoter probe. To evaluate whether H-NS protein bound to the shifted DNA probe decreases and ArcA bound to the shifted DNA probe increases as ArcA concentration increases, EMSA reactions were transferred to nitrocellulose membrane such that the proteins bound to DNA probe could be monitored by immunoblotting. As shown in Fig. 8E, H-NS

protein signal corresponding to the H-NS-*eltA* bound complex diminished whereas ArcA signal intensified as ArcA concentration increased across reactions. Similar results were obtained for *estB* and *hlyC* (Fig. S3). Altogether, these results suggest that ArcA positively regulates *eltA*, *estB* and *hlyC* by counteracting H-NS's repression.

Discussion

The emergence of hybrid Diarrheagenic *Escherichia coli* (DEC) strains, which result from the fusion of genetic markers from different pathotypes, poses a global public health concern (47). This phenomenon is fueled by the presence of numerous virulence markers, often harbored on mobile genetic elements (MGEs) such as phages and plasmids. The mobility of these virulence genes via horizontal gene transfer fosters the evolution of hybrid pathotypes (48-52). STEC and Enterotoxigenic E. coli (ETEC) are significant contributors to diarrhea in both humans and animals globally. Recent reports from diverse geographical regions, including Finland, Bangladesh, Sweden, and South Korea, have revealed the existence of hybrids resulting from the combination of STEC and ETEC strains, categorized as STEC/ETEC hybrids (7, 8, 53-55). Genome-wide phylogenetic analyses have shown a strong association between these hybrids and specific strains of ETEC and STEC, suggesting potential acquisition of Stx-phage and/or ETEC virulence genes during the evolution of STEC/ETEC hybrids (1, 10-12). The pivotal role of Stx-phages in facilitating gene transfer and driving the evolution of E. coli, including the development of STEC variants, cannot be overstated (56). Furthermore, plasmids carrying genes encoding ST and LT toxins have demonstrated their capacity to transfer among diverse E. coli strains (57).

Notably, certain hybrid strains within this category have been epidemiologically linked to diarrheal diseases and Hemolytic Uremic Syndrome (HUS) in humans (7, 8, 54), underscoring the critical need for a comprehensive understanding of their virulence mechanisms. However, there remains a paucity of studies addressing the virulence and regulatory mechanisms of STEC/ETEC hybrid strains isolated from porcine sources. Therefore, this study aims to elucidate the virulence factors expressed by a hybrid ETEC/STEC strain and uncover the underlying pathogenic mechanisms employed by this unique pathogen.

Diarrhea caused by ETEC is very common in farm animals. ETEC strains produce two major types of VFs; adhesins that facilitate binding and colonization of host gut epithelium and enterotoxins that induce fluid secretion (4). To establish a successful infection, expression of VFs needs to be well coordinated. Here, we showed that several major VFs of STEC/ETEC are highly expressed under microaerobiosis, and the response regulator ArcA is required for full expression of the adhesin F18 and several other toxins, including ST and LT. F18 is a predominant adhesin of ETEC causing PWD and plays a crucial role in adherence to host cells (Fig. 3A), therefore deletion of *arcA* impaired the adherence of ETEC to porcine intestinal epithelial cells (Fig. 2E). Among the affected toxins, ST and LT have been shown to affect ETEC virulence and/or colonization in mouse models (58, 59). Indeed, a lack of *arcA* severely reduced ETEC virulence in a mouse model. Further bioinformatic and experimental approaches demonstrate that ArcA antagonizes H-NS's repression of target genes. Therefore, this study described the regulatory details of an important

virulence regulator as well as its contributions to virulence in STEC/ETEC.

Interestingly, expression of the F18 fimbriae was not detectable under laboratory conditions. This is consistent with previous reports that not all strains express F18 *in vitro*. For example, most F18ab fimbriae of clinical isolates are produced at minimal levels on common laboratory media (60). Our results showed that expression of *fed* genes was detectable during *in vivo* infection or bacteria-host interaction. Similarly, a transcriptome study has revealed that several adhesins, such as type 1 fimbriae and a secreted autotransporter EatA, are activated following attachment of ETEC to intestinal epithelial cells (61). These data suggest that expression of ETEC VFs is tightly controlled and stimulated only when needed. However, the regulators involved in this process remain largely unknown. Here we showed that the lack of *arcA* decreased the F18 expression to undetectable levels. Therefore, ArcA serves as a positive contributor in this host-adapted pattern of gene expression.

Besides host contact, oxygen is another important signal for human ETEC to regulate the expression of VFs, such as enterotoxins (32). This was reflected in our results that major ETEC VFs (ST, Stx2e and hemolysin) are expressed at higher levels in microaerobiosis relative to anaerobiosis (Fig. 1). Microaerobic conditions also serve as inducing signals for virulence expression in some other enteric pathogens. In EHEC (62) and *Shigella* (63), type III secretion system (T3SS) can be induced by microaerobic conditions; likewise, in *Salmonella* virulence genes on the pESI virulence plasmid are upregulated under microaerobiosis (64). Further, our results have suggested a positive role of ArcA in virulence regulation under microaerobiosis,

and this role contrasted with the repressor FNR. Using a controlled human infection model for ETEC, Crofts *et al.* suggested that when inside the intestinal lumen where oxygen is very limited, expression of VFs are repressed by FNR. By contrast, as ETEC nears the epithelium, oxygen diffused from the epithelial cells can relieve FNR repression; and subsequently ETEC can colonize the tissue with the derepressed VFs (32). FNR and ArcA co-regulate multiple genes in *E. coli* K-12 (35), it seems highly likely that ArcA and FNR coordinate to optimal expression of virulence genes in ETEC. However, it is noteworthy that signals like salt and glucose concentration (30), and regulator proteins like CRP and H-NS (31) can modulate VF expression at other levels, thereby forming a regulatory network.

We also found that positions of the potential ArcA binding sites relative to the TSS are variable among the positively regulated genes (Figs 3 to 7). These are in line with the findings made by Park *et al.*, where they mapped the ArcA binding sites at a global scale and found that some binding sites are located downstream of TSS, while others occupy the -10 and/or -35 motifs. It has been suggested that activation by ArcA may not be through direct ArcA-RNA polymerase interaction, but rather through antirepression (35). Interestingly, our data indicate that H-NS, a global silencer on genes predominantly acquired through horizontal gene transfer (65), binds to the promoter regions of VFs and represses their expression; and that activation of *eltA*, *hlyC* and *estB* by ArcA is dependent on H-NS. We further demonstrate that ArcA can displace H-NS from the promoter of target genes, thereby alleviating H-NS's repression (Fig. 8). It seems not surprising that ArcA and H-NS compete for similar

sites at a promoter, as both ArcA and H-NS binding sites on DNA are AT-rich sequences (35, 66). Mechanistically, H-NS potentially causes bridge formation on DNA, thus blocking transcription initiation and/or elongation (67, 68); and eviction of H-NS from DNA by ArcA can lead to structural changes, e.g. opening of the complex, allowing transcription to start and continue. Similarly, Ler, a positive regulator of T3SS in enterohemorrhagic *E. coli*, competes for binding to the promoter region against H-NS and activates the expression of T3SS and virulence (44). Hence, our data reveal a previously unrecognized virulence counter-silencing mechanism in ETEC. But in the case of *stx2e*, ArcA positively regulates *stx2e*, independently of H-NS (Fig. S4), and this may be correlated to the distinctive binding site architecture (inverted repeats versus normally directed repeats). The molecular details underlying this regulatory mode warrant more future research.

In conclusion, the present work established that ArcA regulates multiple virulence genes under microaerobiosis and is required for full virulence in cell culture and mouse models. Therefore, ArcA can be added to the growing list of virulence regulators in STEC/ETEC. The discovery of drugs targeting ArcA may serve as therapeutic or prevention against STEC/ETEC caused porcine diarrheal diseases.

Materials and Methods

Bacterial strains, culture conditions, and plasmids.

The wild-type strain STEC/ETEC MD724-020 (O141:H4) was isolated from the small intestine of a nursery-age pig that suffered from diarrhea and acute mortality in Maryland, United States, in May 2014 (69, 70). This particular strain was isolated

through culturing on blood agar plates (5% sheep blood) and subsequently identified using Matrix Assisted Laser Desorption/Ionization Time of Flight Mass Spectrometry (MALDI-TOF MS). Pathotyping of the strain was achieved through whole genome sequencing (WGS), revealing positive results for the presence of key genes including *eltA*, *estB*, *stx2*, *stx2e*, *eastA*, *hlyCABD*, *hlyE*, and the adhesin (F18) gene. The comprehensive WGS data have been deposited in the National Center for Biotechnology Information (NCBI) database under the Sequence Read Archive (SRA) accession SRR7213616. The strains and plasmids used in this study are listed in Table S1. Aerobic growth was achieved by shaking in air at 160 rpm, while anaerobic and microaerobic culturing was conducted using AnaeroPack or MicaeroPack (Mitsubishi Gas Chemical Company, Japan) in a sealed jar. Growth of ETEC strains were measured in triplicate under aerobic, microaerobic and anaerobic conditions for 12 h or 10 h, using a spectrophotometer. For genetic manipulations and *β*-galactosidase assays, all *E. coli* strains were routinely grown in tryptic soy broth (TSB) medium. Selective antibiotics and isopropyl-*β*-D-thiogalactopyranoside (IPTG) were added wherever necessary at the following concentrations: ampicillin, 50 μg/mL; kanamycin, 50 μg/mL; chloramphenicol, 25 μg/mL; nalidixic acid, 30 μg/mL; and IPTG, 0.1 mM.

Recombinant DNA techniques.

PCR, DNA ligation, electroporation, and DNA gel electrophoresis were performed as described by Sambrook and Russell (71), unless otherwise indicated. Oligonucleotide primers were produced in the Iowa State University DNA facility (Iowa, USA) or purchased from BGI Tech Solutions (Guangzhou, China) and are listed in Table S2 in

the Supplemental Material. All restriction and DNA-modifying enzymes were purchased from TaKaRa Biotechnology (TaKaRa, Dalian, China) and used according to the supplier's recommendations. Recombinant plasmids, PCR products, and restriction fragments were purified using TaKaRa MiniBEST plasmid purification kits or agarose gel extraction kits (TaKaRa, Shiga, Japan) as recommended by the supplier. DNA sequencing was performed at the DNA facility of Shanghai Sunny Biotechnology Co., Ltd. Deletion mutants were constructed using the bacteriophage lambda red recombinase system described by Datsenko and Wanner (72). The mutants were confirmed by PCR and DNA sequencing.

Plasmid construction.

For complementation, gene-coding sequences and their putative promoter regions were amplified using the primers listed in Table S2 from the WT strain and independently cloned into pGEN-MCS (73) using *Bam*HI and *Eco*RI. Chromosomal transcriptional *lacZ* fusion was constructed by homologous recombination of the suicidal plasmid pVIK112 carrying a fragment of the complete 5′-region of the target gene (74, 75). Briefly, PCR fragments of target genes were cloned into pVIK112 using *Eco*RI and *Xba*I sites. The resulting pVIK112 derivatives were introduced into STEC/ETEC MD724-020 Δ*lacZ* by conjugation. Conjugants were selected and confirmed by PCR. To construct the plasmid overproducing the ArcA-His_6 fusion protein, a 717-bp fragment containing the coding region of *arcA* was obtained by PCR from genomic DNA with primers carrying codons for 6×His and subsequently cloned into the pET28a (+) vector (Novagen, Madison, WI, USA) using *Bam*HI and *Hind*III

sites. The resultant plasmid contained pET28a -ArcA-His_6 under the control of the T7 promoter. To construct the plasmid overproducing the H-NS-FLAG fusion protein, a 414-bp fragment containing the coding region of *hns* and 24-bp fragment coding for FLAG plus a stop codon was obtained by PCR and subsequently cloned into the pET21a (+) vector (Novagen). Constructs were verified by Sanger sequencing (Comate corp., China).

Mapping of transcription start sites by 5' RACE.

Mapping of TSS was performed as previously described, but with minor modifications (76). Briefly, total RNA was isolated from wild-type MD724-020 after interaction with IPEC-J2 cells for 1 h. Genomic DNA was removed from the extracted RNA using the RNase-Free DNase set (Qiagen, California, CA, USA) and then reverse transcribed to cDNA using reagents supplied in the SMARTer RACE 5'/3' Kit (TaKaRa, Shiga, Japan) following the manufacturer's instructions. Random primers were used for first-strand cDNA synthesis. The obtained cDNA was then used for 5' RACE amplification with Universal Primer A Mix and gene-specific primers (Table S2). The 5' RACE products were cloned into the pRACE vector by in-fusion cloning. The fragments of several clones were sequenced for mapping of the primary transcription.

Purification of ArcA and H-NS proteins.

Escherichia coli BL21 carrying pET28a-ArcA or pET21a-H-NS were grown in 200 mL of LB medium to an OD_{600}=0.6-0.8, and protein expression was induced by adding 0.1 mM IPTG for 10 h at 28 °C, respectively. The ArcA-His_6 fusion protein

was purified as previously reported (77). The *E. coli* H-NS-FLAG protein was purified by Anti-DYKDDDK G1 Affinity Resin (GenScript, Piscataway, NJ, USA) according to the manufacturer's protocol. Briefly, bacterial pellets after induction were resuspended in lysis buffer (50 mM Tris-HCl, pH 8.0, 150 mM NaCl, 1 mM PMSF, 0.2 mg/mL DNase I, 10 mg/ mL lysozyme) and incubated for 20 min, and then sonicated for 15 min. Supernatant was collected after centrifugation and loaded onto a 2 mL Anti-DYKDDDK G1 Affinity Resin column. Protein was eluted from the column using elution buffer (3 M NaCl). Fractions were analyzed by SDS-PAGE to test the presence and purity of H-NS-FLAG protein. Pooled fractions were concentrated using 10 kDa cutoff centrifugal filters (Sartorius) and dialyzed overnight against storage buffer (20 mM Tris–HCl, pH 8.0, 300 mM NaCl, 1 mM EDTA, 5% glycerol). Dialyzed protein was aliquoted and stored at −80° C.

Electrophoretic mobility shift assays (EMSAs).

To study the binding of ArcA to the DNA probe, EMSAs were performed as described previously. Briefly, DNA probes were amplified using specific primers and purified using a TaKaRa Mini-BEST gel extraction kit. EMSAs were performed by adding increasing amounts of purified and phosphorylated His_6-ArcA fusion protein (0 to 3.5 μM) to the DNA probe (0.1 pmol) in binding buffer (10 mM Tris-HCl, 7 mM $MgCl_2$, 5% glycerol, 40 μg/mL bovine serum albumin, pH 8.0) for 30 min at 37°C. The reaction mixtures were then subjected to electrophoresis on a 6% polyacrylamide gel in 0.5× TBE buffer (44.5 mM Tris, 44.5 mM boric acid, 1 mM EDTA, pH 8.0) at 200 V for 45 min. The gel was stained in 0.5× TBE buffer containing 1× SYBR™ Gold

nucleic acid-staining solution (Life Technologies, Grand Island, NY, USA) for 15 min, and then the image was recorded. To study the binding of H-NS to the DNA probe, binding reactions were performed in EMSA buffer (10 mM Tris-HCl, 50 mM KCl, 5 mM $MgCl_2$, 1 mM DTT, 1 mM EDTA, 5% glycerol, pH 8.0). Reactions were incubated at 37° C for 30 min. For competitive binding experiments, these reactions were then supplemented with the other protein (ArcA) in binding buffer and incubated at 37° C for an additional 15 min. The reaction mixtures were then subjected to electrophoresis on two separate 6% polyacrylamide gel at 200 V for 45 min using identical procedures. DNA was detected by staining the gels with 1× SYBR Gold nucleic acid-staining solution for 15 min at room temperature. To detect H-NS and ArcA proteins in competitive EMSAs, each stained gel was transferred to a nitrocellulose membrane. Proteins were then detected using anti-His-KPL (Sigma) and anti-FLAG-KPL (Sigma) antibodies.

***β*-galactosidase activity.**

Expression levels of the *gene-lacZ* reporter fusions were determined by measuring *β*-galactosidase activity of exponentially growing cultures at 37 °C under microaerobic conditions in TSB medium. Overnight TSB cultures of *E. coli* containing the fusions of the gene of interest with *lacZ* were washed with phosphate-buffered saline, diluted 1:100 in TSB medium and grown at 37 °C to log phase or stationary phase. These cultures were diluted 1:1 in Z buffer and assayed for *β*-galactosidase activity using ortho-nitrophenyl-*β*-galactoside (ONPG) as the substrate as described previously (78). The values of *β*-galactosidase activity were

measured at least in triplicate for each experiment.

Western blot.

Bacteria were cultured in TSB under microaerobic conditions at 37 ℃ as described above and pelleted by centrifugation at 12000 ×g for 2 min. For EltA and Stx2e, cell pellets were resuspended in PBS and subjected to sonication and then centrifuged at 5000 ×g for 10 min to collect the supernatant. For HlyA, Cell pellets were resuspended in double distilled water and mixed with loading buffer. After being lysed by boiling for 10 min, cell lysates were centrifuged at 12000 ×g for 2 min at 4°C. Then, the supernatant was subjected to SDS–PAGE. Proteins were transferred to a polyvinylidene fluoride (PVDF) membrane (Millipore), and the membrane was blocked with 5% skim milk at 37° C for 2 hours. Blots were probed with a polyclonal antibody against EltA (Abcam, ab243102), Stx2e (Abmart, China) HlyA (Abclonal, China) or against GroEL (Abcam, ab90522) and an anti-rabbit IgG KPL DyLight 800-labeled antibody (SeraCare, 5230-0346). Membranes were viewed by using an Odyssey CLx imager (Odyssey LICOR, USA).

STb-Enzyme-linked immunosorbent assay (STb-ELISA).

The level of STb in cell-free culture supernatants was determined by STb-ELISA performed essentially as described previously with minor modifications (79). Briefly, crude culture supernatants (50 μL) for each strain from the BIH-CA media were diluted 1:2 in 0.1 M sodium carbonate buffer (pH 9.6) and used to coat the wells of 96-well flat bottom microtiter plates (Corning Incorporated, Kennebunk, ME). Each strain was coated onto 6 wells, and wells that received only carbonate buffer served as

background control wells. The plates were coated overnight at 4 °C, after which the overnight coating solution was removed, and the plates were washed three times with 0.05% PBS and tapped dry. The remaining unbound reaction sites were blocked by the addition of 200 μL 5% skim milk to all wells and incubated for 1 h at 37° C. After removing the blocking solution and washing plates three times with PBS, 100 μL of mouse anti-STb toxin antibody (Magic Anti-*E. coli* Heat Stable Enterotoxin Monoclonal antibody, Clone N230630) was added to all wells, and plates were incubated for 1 hour at 37° C. The plates were washed three times with PBS, tapped dry and 200 μL of goat anti-mouse IgG alkaline phosphatase conjugate (1:2000 in PBS; Abcam) was added to all wells and incubated for 1 h at 37°C. The plates were again washed three times with PBS and tapped dry. Then, 50 μL of 3,3',5,5'-tetramethylbenzidine (TMB) (Sigma–Aldrich, Shanghai, China) was added to all wells, and the plates were incubated in the dark at 37° C for 15 min. The reaction was stopped by the addition of 50 μL of 2 M H_2SO_4. The optical density of each reaction mix was measured at 450 nm with a plate reader (BioTek, Winooski, VT, USA).

Hemolysis Assay:

To assess hemolysis on blood agar, we followed the procedure outlined in Velasco et al., 2018. Briefly, we streaked the WT, Δ*hlyA*, and Δ*hlyE* strains onto blood agar plates containing 10% defibrinated sheep blood. These plates were then incubated at 37°C for 18–24 hours under microaerobic conditions. The extent of hemolysis was determined by the size and translucence of the hemolytic halo—larger or more

translucent halos indicated stronger hemolytic activity, while smaller or less translucent halos indicated weaker activity.

Hemagglutination Assay:

To evaluate the bacterial cell surface expression of F18 fimbriae, we employed a hemagglutination assay using the WT, Δ*arcA* mutant, and Δ*arcA* complemented strains. This assay utilized fresh bacterial suspensions and chicken red blood cells, with a negative control consisting of a saline solution (0.9% NaCl). The hemagglutination tests were conducted in V-bottom microtiter plates. We prepared a series of 2-fold dilutions of the bacterial isolates in separate microtiter wells using saline solution. To each well containing the bacterial dilutions, we added equal volumes of washed red blood cells (RBCs). After gently mixing, the microtiter plate was incubated at 37°C for 1 hour.

Adherence assays.

Cell adherence assays were performed as previously described (80). The neonatal jejunal epithelial cell line IPEC-J2 was cultured in Dulbecco's Minimal Essential Medium (DMEM) basic (Gibco, Grand Island, NY, USA) containing 10% fetal bovine serum (FBS; Gibco, Grand Island, NY, USA) at 37 °C in 5% CO_2. Cells were plated in sterile 24-well plates at 6×10^4 cells/well 24 h before the experiment. STEC/ETEC MD724-020 wild-type, Δ*arcA* mutant, and complemented strains were cultured statically in TSB medium until the OD_{600} was 0.6. Before infection, two wells of cultured cells were trypsinized, and the cells were counted to estimate the cell number per well. Cells were washed twice with 1 ml PBS and were then exposed to

bacteria at a multiplicity of infection (MOI) of 10 and washed once with PBS. The 24-well plates were centrifuged (500 ×g for 5 min) and incubated for 1 h. For the adherence assays, bacterium-exposed cells were washed four times with 1 ml of sterile PBS and then lysed with 1 ml 0.1% Triton X-100 for 5 min at room temperature. Serial dilutions of cell suspensions were spread onto MacConkey agar plates (Becton Dickinson & Co, Franklin Lakes, NJ), and CFU counts were obtained after overnight growth at 37 °C. The input dilution of bacteria was also plated to determine the CFU count for each inoculum.

Mouse infection by ETEC.

Healthy 4-week-old BALB/c mice were used as the ETEC infection model (31, 81). All animals were allowed free access to sterile water and animal feed and were handled according to the guidelines of the Laboratory Animal Monitoring Committee of Heilongjiang Province. Mice were randomly divided into three groups according to the experiment with four female and four male mice in each group. In this study, the three groups were challenged with WT, Δ*arcA* mutant or Δ*arcA* complementation strains. Following a 2-day acclimation period upon arrival, mice were given gentamicin (35 mg/L), vancomycin (45 mg/L), metronidazole (215 mg/L), and colistin (850 U/mL) in drinking water to disrupt resident microbiota as previously published (82). After 3 days on antibiotics in water, mice were given untreated water for 1 day. Each mouse was weighed and recorded 0 days before infection and then given a single oral challenge by gavage of 1×10^9 CFU ETEC in 100 μL of a 1.2% carbonate solution. Feces were collected from infected mice for three days, and the

fecal bacterial load of each mouse was detected by blood agar plate counting. After the challenge, mice were monitored and weighed daily for a total of two weeks. When the animal test was completed, alive mice were humanely euthanized.

Histology.

Mice were grouped and challenged with ETEC strains as described above. At 24 h post infection, mice were sacrificed to collect the jejunum and ileum. Tissues were fixed with 4% paraformaldehyde and stained with hematoxylin and eosin (H&E).

RT–PCR and quantitative real-time RT–PCR.

RT–PCR was used for the co-transcription test. RNA of the wild-type MD724-020 during interaction with IPEC-J2 cells was extracted using a RNeasy Mini Kit (Qiagen) with one-hour in-tube DNase digestion (Qiagen) to remove possible DNA contamination according to the manufacturer's instructions. One microgram of total RNA was reverse transcribed in triplicate using random hexamers and ImProm-II reverse transcriptase (Promega, Madison, WI, USA). For RT–PCR, primer pairs were designed to span adjacent genes. cDNA was then used as the template for subsequent PCRs, and RNA that was not reverse transcribed was used as a negative control.

Real-time quantitative PCR (RT–qPCR) was used to validate the expression levels of selected genes. For the *in vivo* mouse infection test, total RNA was extracted from the intestinal contents of mice infected with WT, Δ*arcA* mutant or Δ*arcA* complementation strains using the E.Z.N.A Stool RNA Kit (Omega, Norcross, GA, USA), and non-infected mice were included as a control. For the *in vitro* cell infection test, IPEC-J2 cells were previously infected with WT, Δ*arcA* mutant or Δ*arcA*

complementation strains for one hour. Then, the medium was removed, and the cells were washed gently three times with PBS and resuspended in 0.1% PBST. The mixture of cells and bacteria was centrifuged for 5 min at ×1000 g, the supernatant was collected and centrifuged for 2 min at ×12 000 g, and then the collected bacteria were used for RNA extraction using the SV Total RNA Isolation System (Promega, Madison, WI, USA). Total RNA from *in vivo* and *in vitro* experiments was treated with gDNA Eraser and reverse transcribed with random primers and reverse transcriptase using the PrimerScript^{TM} RT reagent Kit with gDNA Eraser (TaKaRa). The primer pairs used are listed in Table S1 in the supplemental material, and RT–qPCR was performed as previously described (77, 83). Melting-curve analyses were performed after each reaction to ensure amplification specificity. The Ct value was set as 38 when gene expression was below detectable level. Differences (*n*-fold) in transcripts were calculated using the relative comparison method, and the amplification efficacies of each primer set were verified as described by Schmittgen *et al.* (84). RNA levels were normalized using the housekeeping gene *tus* for the replication terminator protein as a control.

Statistical analysis.

Figures were plotted with GraphPad Prism 8 software. The significance of differences between groups was evaluated using SPASS statistics version 20 (SPASS Software). One-way ANOVA was used when the sample size was greater than two, and Student's *t* test was applied for two-group analysis. Differences were considered significant at a *P* value of 0.05.

Ethics statement.

Handling and Care of mice were performed according to the Beijing Administration Guidelines for the Use of Laboratory Animals. The entire protocol with respect to animal experiments was approved by the Review Board of Harbin Veterinary Research Institute and by the Animal Care and Use Committee of Heilongjiang Province (SYXK(H)2006–032). All efforts were made to minimize suffering of animals.

Acknowledgments

This work was supported by National Natural Science Foundation of China Young Scholars Project (Grant number: 32002257). The funders played no roles in study design, data collection and interpretation, or submission for publication.

G.-W.L. and W.-T.C. conceived, designed, coordinated the study and revised the manuscript. F.-W.J. carried out most of the experiments, analyzed the data, and wrote the initial version of the manuscript. Y.Y. and Z.M. assisted with animal experiments. All authors provided input to, read, and approved the final manuscript.

All authors declared that they have no conflicts of interests.

References

1. Anderson JDt, Bagamian KH, Muhib F, Amaya MP, Laytner LA, Wierzba T, Rheingans R. 2019. Burden of enterotoxigenic *Escherichia coli* and *shigella* non-fatal diarrhoeal infections in 79 low-income and lower middle-income countries: a modelling analysis. Lancet Glob Health 7:e321-e330.

2. Bagamian KH, Anderson JDt, Muhib F, Cumming O, Laytner LA, Wierzba TF, Rheingans R. 2020. Heterogeneity in enterotoxigenic *Escherichia coli* and *shigella* infections in children under 5 years of age from 11 African countries: a subnational approach quantifying risk, mortality, morbidity, and stunting. Lancet Glob Health 8:e101-e112.

3. Levine MM. 1981. Adhesion of enterotoxigenic *Escherichia coli* in humans and animals. Ciba Foundation symposium 80:142-160.

4. Dubreuil JD, Isaacson RE, Schifferli DM. 2016. Animal Enterotoxigenic *Escherichia coli*. EcoSal Plus 7.

5. Fairbrother JM, Nadeau E, Gyles CL. 2005. *Escherichia coli* in postweaning diarrhea in pigs: an update on bacterial types, pathogenesis, and prevention strategies. Anim Health Res Rev 6:17-39.

6. Zhang W, Zhao M, Ruesch L, Omot A, Francis D. 2007. Prevalence of virulence genes in *Escherichia coli* strains recently isolated from young pigs with diarrhea in the US. Vet Microbiol 123:145-52.

7. Nyholm O, Heinikainen S, Pelkonen S, Hallanvuo S, Haukka K, Siitonen A. 2015. Hybrids of Shigatoxigenic and Enterotoxigenic *Escherichia coli*

(STEC/ETEC) Among Human and Animal Isolates in Finland. Zoonoses Public Health 62:518-24.

8. Bai X, Zhang J, Ambikan A, Jernberg C, Ehricht R, Scheutz F, Xiong Y, Matussek A. 2019. Molecular Characterization and Comparative Genomics of Clinical Hybrid Shiga Toxin-Producing and Enterotoxigenic *Escherichia coli* (STEC/ETEC) Strains in Sweden. Sci Rep 9:5619.

9. Fratamico PM, Bagi LK, Bush EJ, Solow BT. 2004. Prevalence and characterization of shiga toxin-producing *Escherichia coli* in swine feces recovered in the National Animal Health Monitoring System's Swine 2000 study. Appl Environ Microbiol 70:7173-8.

10. da Silva AS, Valadares GF, Penatti MP, Brito BG, da Silva Leite D. 2001. *Escherichia coli* strains from edema disease: O serogroups, and genes for Shiga toxin, enterotoxins, and F18 fimbriae. Vet Microbiol 80:227-33.

11. Osek J. 2000. Virulence factors and genetic relatedness of *Escherichia coli* strains isolated from pigs with post-weaning diarrhea. Vet Microbiol 71:211-22.

12. Baldo V, Salogni C, Giovannini S, D'Incau M, Boniotti MB, Birbes L, Pitozzi A, Formenti N, Grassi A, Pasquali P, Alborali GL. 2020. Pathogenicity of Shiga Toxin Type 2e *Escherichia coli* in Pig Colibacillosis. Front Vet Sci 7:545818.

13. Brilhante M, Perreten V, Dona V. 2019. Multidrug resistance and multivirulence plasmids in enterotoxigenic and hybrid Shiga

toxin-producing/enterotoxigenic *Escherichia coli* isolated from diarrheic pigs in Switzerland. Vet J 244:60-68.

14. Kusumoto M, Hikoda Y, Fujii Y, Murata M, Miyoshi H, Ogura Y, Gotoh Y, Iwata T, Hayashi T, Akiba M. 2016. Emergence of a Multidrug-Resistant Shiga Toxin-Producing Enterotoxigenic *Escherichia coli* Lineage in Diseased Swine in Japan. J Clin Microbiol 54:1074-81.

15. Nagy B, Casey TA, Moon HW. 1990. Phenotype and genotype of *Escherichia coli* isolated from pigs with postweaning diarrhea in Hungary. J Clin Microbiol 28:651-3.

16. Moon HW, Hoffman LJ, Cornick NA, Booher SL, Bosworth BT. 1999. Prevalences of some virulence genes among *Escherichia coli* isolates from swine presented to a diagnostic laboratory in Iowa. J Vet Diagn Invest 11:557-60.

17. Smeds A, Hemmann K, Jakava-Viljanen M, Pelkonen S, Imberechts H, Palva A. 2001. Characterization of the adhesin of *Escherichia coli* F18 fimbriae. Infect Immun 69:7941-5.

18. Lu T, Seo H, Moxley RA, Zhang W. 2019. Mapping the neutralizing epitopes of F18 fimbrial adhesin subunit FedF of enterotoxigenic *Escherichia coli* (ETEC). Vet Microbiol 230:171-177.

19. Tiels P, Verdonck F, Smet A, Goddeeris B, Cox E. 2005. The F18 fimbrial adhesin FedF is highly conserved among F18+ *Escherichia coli* isolates. Vet Microbiol 110:277-83.

20. Moonens K, Bouckaert J, Coddens A, Tran T, Panjikar S, De Kerpel M, Cox E, Remaut H, De Greve H. 2012. Structural insight in histo-blood group binding by the F18 fimbrial adhesin FedF. Mol Microbiol 86:82-95.

21. Spangler BD. 1992. Structure and function of cholera toxin and the related *Escherichia coli* heat-labile enterotoxin. Microbiol Rev 56:622-47.

22. Nataro JP, Kaper JB. 1998. Diarrheagenic *Escherichia coli*. Clinical microbiology reviews 11:142-201.

23. Kennedy D, Greenberg R, Dunn J, Abernathy R, Ryerse J, Guerrant R. 1984. Effects of *Escherichia coli* heat-stable enterotoxin STb on intestines of mice, rats, rabbits, and piglets. Infect Immun 46:639-643.

24. Gill DM, Richardson SH. 1980. Adenosine diphosphate-ribosylation of adenylate cyclase catalyzed by heat-labile enterotoxin of *Escherichia coli*: comparison with cholera toxin. J Infect Dis 141:64-70.

25. Yoshimura S, Ikemura H, Watanabe H, Aimoto S, Shimonishi Y, Hara S, Takeda T, Miwatani T, Takeda Y. 1985. Essential structure for full enterotoxigenic activity of heat-stable enterotoxin produced by enterotoxigenic *Escherichia coli*. FEBS letters 181:138-142.

26. Guth BE. 2000. Enterotoxigenic *Escherichia coli*--an overview. Memorias do Instituto Oswaldo Cruz 95 Suppl 1:95-97.

27. Ludwig A, von Rhein C, Bauer S, Hüttinger C, Goebel W. 2004. Molecular analysis of cytolysin A (ClyA) in pathogenic *Escherichia coli* strains. J Bacteriol 186:5311-5320.

28. Rennie R, Freer J, Arbuthnott J. 1974. The kinetics of erythrocyte lysis by *Escherichia coli* haemolysin. J Med Microbiol 7:189-195.

29. Faubert C, Drolet R. 1992. Hemorrhagic gastroenteritis caused by *Escherichia coli* in piglets: Clinical, pathological and microbiological findings. Can Vet J 33:251-6.

30. Munson GP. 2013. Virulence regulons of enterotoxigenic *Escherichia coli*. Immunol Res 57:229-36.

31. Haycocks JR, Sharma P, Stringer AM, Wade JT, Grainger DC. 2015. The molecular basis for control of ETEC enterotoxin expression in response to environment and host. PLoS Pathog 11:e1004605.

32. Crofts AA, Giovanetti SM, Rubin EJ, Poly FM, Gutiérrez RL, Talaat KR, Porter CK, Riddle MS, DeNearing B, Brubaker J, Maciel M, Jr., Alcala AN, Chakraborty S, Prouty MG, Savarino SJ, Davies BW, Trent MS. 2018. Enterotoxigenic *E. coli* virulence gene regulation in human infections. Proc Natl Acad Sci U S A 115:E8968-e8976.

33. Lynch AS, Lin EC. 1996. Transcriptional control mediated by the ArcA two-component response regulator protein of *Escherichia coli*: characterization of DNA binding at target promoters. J Bacteriol 178:6238-49.

34. Bekker M, Alexeeva S, Laan W, Sawers G, de Mattos JT, Hellingwerf K. 2010. The ArcBA Two-Component System of *Escherichia coli* Is Regulated by the Redox State of both the Ubiquinone and the Menaquinone Pool. J Bacteriol 192:746-754.

35. Park DM, Akhtar MS, Ansari AZ, Landick R, Kiley PJ. 2013. The bacterial response regulator ArcA uses a diverse binding site architecture to regulate carbon oxidation globally. PLoS Genet 9:e1003839.

36. Salmon KA, Hung S-p, Steffen NR, Krupp R, Baldi P, Hatfield GW, Gunsalus RP. 2005. Global Gene Expression Profiling in *Escherichia coli* K12 effects of oxygen availability and ArcA. J Biol Chem 280:15084-15096.

37. Oshima T, Aiba H, Masuda Y, Kanaya S, Sugiura M, Wanner BL, Mori H, Mizuno T. 2002. Transcriptome analysis of all two-component regulatory system mutants of *Escherichia coli* K-12. Mol Microbiol 46:281-91.

38. He G, Shankar RA, Chzhan M, Samouilov A, Kuppusamy P, Zweier JL. 1999. Noninvasive measurement of anatomic structure and intraluminal oxygenation in the gastrointestinal tract of living mice with spatial and spectral EPR imaging. Proc Natl Acad Sci U S A 96:4586-91.

39. Nagy B, Wilson RA, Whittam TS. 1999. Genetic diversity among *Escherichia coli* isolates carrying f18 genes from pigs with porcine postweaning diarrhea and edema disease. J Clin Microbiol 37:1642-5.

40. Münch R, Hiller K, Grote A, Scheer M, Klein J, Schobert M, Jahn D. 2005. Virtual Footprint and PRODORIC: an integrative framework for regulon prediction in prokaryotes. Bioinformatics 21:4187-4189.

41. Ludwig A, von Rhein C, Bauer S, Huttinger C, Goebel W. 2004. Molecular analysis of cytolysin A (ClyA) in pathogenic *Escherichia coli* strains. J Bacteriol 186:5311-20.

42. Meng J, Zhao S, Doyle MP. 1998. Virulence genes of Shiga toxin-producing *Escherichia coli* isolated from food, animals and humans. Int J Food Microbiol 45:229-35.

43. Chaparian RR, Tran MLN, Miller Conrad LC, Rusch DB, van Kessel JC. 2020. Global H-NS counter-silencing by LuxR activates quorum sensing gene expression. Nucleic Acids Res 48:171-183.

44. Mellies JL, Barron AM, Carmona AM. 2007. Enteropathogenic and enterohemorrhagic *Escherichia coli* virulence gene regulation. Infect Immun 75:4199-210.

45. Govantes F, Orjalo AV, Gunsalus RP. 2000. Interplay between three global regulatory proteins mediates oxygen regulation of the *Escherichia coli* cytochrome d oxidase (cydAB) operon. Mol Microbiol 38:1061-73.

46. Atlung T, Sund S, Olesen K, Brøndsted L. 1996. The histone-like protein H-NS acts as a transcriptional repressor for expression of the anaerobic and growth phase activator AppY of *Escherichia coli*. J Bacteriol 178:3418-25.

47. Santos ACM, Santos FF, Silva RM, Gomes TAT. 2020. Diversity of Hybrid- and Hetero-Pathogenic *Escherichia coli* and Their Potential Implication in More Severe Diseases. Front Cell Infect Microbiol 10:339.

48. Kaper JB, Nataro JP, Mobley HL. 2004. Pathogenic *Escherichia coli*. Nat Rev Microbiol 2:123-40.

49. Croxen MA, Finlay BB. 2010. Molecular mechanisms of *Escherichia coli* pathogenicity. Nat Rev Microbiol 8:26-38.

50. Leimbach A, Hacker J, Dobrindt U. 2013. E. coli as an all-rounder: the thin line between commensalism and pathogenicity. Curr Top Microbiol Immunol 358:3-32.

51. Johnson JR, Russo TA. 2018. Molecular Epidemiology of Extraintestinal Pathogenic *Escherichia coli*. EcoSal Plus 8.

52. Lee W, Kim E, Zin H, Sung S, Woo J, Lee MJ, Yang SM, Kim SH, Kim SH, Kim HY. 2022. Genomic characteristics and comparative genomics analysis of *Salmonella enterica s*ubsp. enterica serovar Thompson isolated from an outbreak in South Korea. Sci Rep 12:20553.

53. Johura FT, Parveen R, Islam A, Sadique A, Rahim MN, Monira S, Khan AR, Ahsan S, Ohnishi M, Watanabe H, Chakraborty S, George CM, Cravioto A, Navarro A, Hasan B, Alam M. 2016. Occurrence of Hybrid *Escherichia coli* Strains Carrying Shiga Toxin and Heat-Stable Toxin in Livestock of Bangladesh. Front Public Health 4:287.

54. Oh KH, Shin E, Jung SM, Im J, Cho SH, Hong S, Yoo CK, Chung GT. 2017. First Isolation of a Hybrid Shigatoxigenic and Enterotoxigenic *Escherichia coli* Strain Harboring the stx2 and elt Genes in Korea. Jpn J Infect Dis 70:347-348.

55. Lee W, Kim MH, Sung S, Kim E. 2023. Genome-Based Characterization of Hybrid Shiga Toxin-Producing and Enterotoxigenic *Escherichia coli* (STEC/ETEC) Strains Isolated in South Korea, 2016-2020. Microorganisms 11:1285.

56. Schmidt H. 2001. Shiga-toxin-converting bacteriophages. Res Microbiol 152:687-95.

57. Turner SM, Chaudhuri RR, Jiang ZD, DuPont H, Gyles C, Penn CW, Pallen MJ, Henderson IR. 2006. Phylogenetic comparisons reveal multiple acquisitions of the toxin genes by enterotoxigenic *Escherichia coli* strains of different evolutionary lineages. J Clin Microbiol 44:4528-36.

58. Allen KP, Randolph MM, Fleckenstein JM. 2006. Importance of heat-labile enterotoxin in colonization of the adult mouse small intestine by human enterotoxigenic *Escherichia coli* strains. Infect Immun 74:869-75.

59. Bertin A. 1983. Virulence factors of enterotoxigenic *E. coli* studied in the infant mouse model. Ann Rech Vet 14:169-82.

60. Nagy B, Whipp SC, Imberechts H, Bertschinger HU, Dean-Nystrom EA, Casey TA, Salajka E. 1997. Biological relationship between F18ab and F18ac fimbriae of enterotoxigenic and verotoxigenic *Escherichia coli* from weaned pigs with oedema disease or diarrhoea. Microb Pathog 22:1-11.

61. Kansal R, Rasko DA, Sahl JW, Munson GP, Roy K, Luo Q, Sheikh A, Kuhne KJ, Fleckenstein JM. 2013. Transcriptional modulation of enterotoxigenic *Escherichia coli* virulence genes in response to epithelial cell interactions. Infect Immun 81:259-70.

62. Schuller S, Phillips AD. 2010. Microaerobic conditions enhance type III secretion and adherence of enterohaemorrhagic *Escherichia coli* to polarized human intestinal epithelial cells. Environ Microbiol 12:2426-35.

63. Marteyn B, West NP, Browning DF, Cole JA, Shaw JG, Palm F, Mounier J, Prevost MC, Sansonetti P, Tang CM. 2010. Modulation of *Shigella* virulence in response to available oxygen in vivo. Nature 465:355-8.

64. Aviv G, Rahav G, Gal-Mor O. 2016. Horizontal Transfer of the Salmonella enterica Serovar Infantis Resistance and Virulence Plasmid pESI to the Gut Microbiota of Warm-Blooded Hosts. mBio 7.

65. Singh K, Milstein JN, Navarre WW. 2016. Xenogeneic Silencing and Its Impact on Bacterial Genomes. Annu Rev Microbiol 70:199-213.

66. Gordon BR, Li Y, Cote A, Weirauch MT, Ding P, Hughes TR, Navarre WW, Xia B, Liu J. 2011. Structural basis for recognition of AT-rich DNA by unrelated xenogeneic silencing proteins. Proc Natl Acad Sci U S A 108:10690-5.

67. Dame RT, Noom MC, Wuite GJ. 2006. Bacterial chromatin organization by H-NS protein unravelled using dual DNA manipulation. Nature 444:387-90.

68. Kotlajich MV, Hron DR, Boudreau BA, Sun Z, Lyubchenko YL, Landick R. 2015. Bridged filaments of histone-like nucleoid structuring protein pause RNA polymerase and aid termination in bacteria. Elife 4.

69. Jiang F, Wu Z, Zheng Y, Frana TS, Sahin O, Zhang Q, Li G. 2019. Genotypes and Antimicrobial Susceptibility Profiles of Hemolytic *Escherichia coli* from Diarrheic Piglets. Foodborne Pathog Dis 16:94-103.

70. Wang M, Zeng Z. 2020. Role of enterotoxigenic *Escherichia coli* prophage in spreading antibiotic resistance in a porcine-derived environment. Environ

Microbiol 22:4974-4984.

71. Joseph S, David WR. 2001. Molecular cloning: a laboratory manual. Gold Spring Harbor, New York.

72. Datsenko KA, Wanner BL. 2000. One-step inactivation of chromosomal genes in *Escherichia coli* K-12 using PCR products. Proc Natl Acad Sci U S A 97:6640-6645.

73. Lane MC, Alteri CJ, Smith SN, Mobley HL. 2007. Expression of flagella is coincident with uropathogenic *Escherichia coli* ascension to the upper urinary tract. Proc Natl Acad Sci U S A 104:16669-16674.

74. Kalogeraki VS, Winans SC. 1997. Suicide plasmids containing promoterless reporter genes can simultaneously disrupt and create fusions to target genes of diverse bacteria. Gene 188:69-75.

75. Cai W, Wannemuehler Y, Dell'Anna G, Nicholson B, Barbieri NL, Kariyawasam S, Feng Y, Logue CM, Nolan LK, Li G. 2013. A novel two-component signaling system facilitates uropathogenic *Escherichia coli*'s ability to exploit abundant host metabolites. PLoS Pathog 9:e1003428.

76. Li H, Hu S, Yan X, Yang Y, Liu W, Bu Z, Li G, Cai W. 2021. An extracytoplasmic function (ECF) sigma/anti-sigma factor system regulates hypochlorous acid resistance and impacts expression of the type IV secretion system in *Brucella melitensis*. J Bacteriol doi:10.1128/JB.00127-21.

77. Jiang F, An C, Bao Y, Zhao X, Jernigan RL, Lithio A, Nettleton D, Li L, Wurtele ES, Nolan LK, Lu C, Li G. 2015. ArcA Controls Metabolism,

Chemotaxis, and Motility Contributing to the Pathogenicity of Avian Pathogenic *Escherichia coli*. Infect Immun 83:3545-54.

78. Miller J. 1972. Assay of β-galactosidase. Experiments in molecular genetics:352-355.

79. Erume J, Berberov EM, Moxley RA. 2010. Comparison of the effects of different nutrient media on production of heat-stable enterotoxin-b by *Escherichia coli*. Vet Microbiol 144:160-5.

80. Zhu J, Yin X, Yu H, Zhao L, Sabour P, Gong J. 2011. Involvement of quorum sensing and heat-stable enterotoxin a in cell damage caused by a porcine enterotoxigenic *Escherichia coli* strain. Infect Immun 79:1688-95.

81. Byrd W, Mog SR, Cassels FJ. 2003. Pathogenicity and immune response measured in mice following intranasal challenge with enterotoxigenic *Escherichia coli* strains H10407 and B7A. Infect Immun 71:13-21.

82. Bolick DT, Kolling GL, Moore JH, 2nd, de Oliveira LA, Tung K, Philipson C, Viladomiu M, Hontecillas R, Bassaganya-Riera J, Guerrant RL. 2014. Zinc deficiency alters host response and pathogen virulence in a mouse model of enteroaggregative *Escherichia coli*-induced diarrhea. Gut Microbes 5:618-27.

83. Mushtaq S, Irfan S, Sarma J, Doumith M, Pike R, Pitout J, Livermore D, Woodford N. 2011. Phylogenetic diversity of *Escherichia coli* strains producing NDM-type carbapenemases. J Antimicrob Chemothe 66:2002-2005.

84. Bustin SA. 2000. Absolute quantification of mRNA using real-time reverse transcription polymerase chain reaction assays. J Mol Endocrinol 25:169-93.

Figure legends

Fig. 1 Microaerobic conditions enhanced the expression of enterotoxin and hemolysin genes. RT–qPCR analysis was performed to examine the expression of *eltA*, *estB*, *stx2e*, *hlyC*, and *hlyE* under microaerobic and anaerobic conditions. Relative expression levels were calculated using the *tus* gene as an internal control, and levels under anaerobic conditions were set as 1.0. Values represent the mean ± standard deviation of triplicate samples from three independent experiments. Significant differences were evaluated by Student's *t* test, and the asterisks indicate **, $P < 0.01$ and *, $P < 0.05$.

Fig. 2 ArcA contributes to the STEC/ETEC virulence. Groups of 8 antibiotic-treated mice were challenged via oral gavage with 10^9 CFU of the wild type the Δ*arcA* mutant or the Δ*arcA* complemented strains, and the survival (A), body weight change (B), fecal bacteria shedding (C) and intestine histopathology (D) were determined at different times post-infection. Data are presented as the mean ± standard deviation. Significant differences were evaluated by log rank test (A) and Student's *t* test (B and C), and the asterisks indicate *, $P < 0.05$, **, $P < 0.01$, and ***, $P < 0.001$. ns, not significant. H&E staining images are representative of three replicates (magnification = 200). (E) Deletion of *arcA* significantly decreased ETEC adherence to IPEC-J2 cells. IPEC-J2 neonatal jejunal epithelial cells were infected with ETEC strains at a multiplicity of infection (MOI) of 10 for 1 h. After 4 washes with PBS, bacteria associated with the cells were spread onto MacConkey agar plates and enumerated. Data are presented as the mean ± standard deviation of triplicate

samples from three independent experiments. Significant differences were evaluated by one-way ANOVA, and the asterisks indicate **, *P* <0.01.

Fig. 3 ArcA positively regulated the expression of *fedA*. (A) Deletion of *fedABCDEF* significantly reduces ETEC adherence to porcine intestinal cells. (B) RT–PCR analysis indicates that the *fedABCDEF* genes are cotranscribed. RNA and cDNA samples were prepared as described above. Primers were designed to span open reading frames (ORFs) of *fedA* and *fedBC*, *fedBC* and *fedD*, *fedD* and *fedE*, and *fedE* and *fedF*, respectively. The black bars under the gene arrows denote the expected PCR amplicons, and the number in front of each bar corresponds to the lane in the agarose gel. The #1 fragment was used as a negative control reaction, as *fedA* and the gene encoding IS66 family transposase are not expected to cotranscribe. RNA that was not reverse transcribed served as a negative control template, while genomic DNA served as a positive control template. (C) Features of the *fedA* promoter region. Italic and bold, start codon; bold, transcription start site; shaded, -35 and -10 regions; and underlined, predicted ArcA binding sites. (D) Production of F18 was validated in WT, Δ*arcA* mutant, Δ*arcA* complemented by chicken hemagglutination assay. (E) Expression levels of *fedA* during ETEC adhering to IPEC-J2 cells were assessed by RT–qPCR. (F) Expression of *fedA* during infection in the mouse intestine was compared in WT, Δ*arcA* and Δ*arcA* complemented strains by RT–qPCR. Relative expression levels were calculated compared with the *tus* gene as an internal control, fold changes are relative to the WT. Data are presented as the mean ± standard deviation of triplicate samples from three independent experiments or from a pool of

three mice. Significant differences were evaluated by one-way ANOVA, and the asterisks indicate **, $P < 0.01$ and *, $P < 0.05$. (G) Nonradioactive electrophoretic mobility shift assay (EMSA) showing phosphorylated ArcA-His_6 binding to the *fedA* promotor region. The PCR product of the *fedA* promoter region containing the ArcA binding site was used as a probe at 0.1 pmol per reaction mixture. A *fedA*-coding region fragment was used as a negative control. DNA fragments were stained with SYBR Gold.

Fig. 4 ArcA positively regulated *eltA* expression. (A) Features of the *eltA* promoter region. Italic and bold, start codon; bold, transcription start site; shaded, -35 and -10 regions; and underlined, predicted ArcA binding sites. (B, C, D and E) A lack of *arcA* led to downregulation of *eltA* in media, during ETEC-host cell interaction and *in vivo*. (B) Expression levels of *eltA* were assessed by measuring *β*-galactosidase activities in *eltA-lacZ* transcriptional fusion strains grown microaerobically at 37 °C in TSB medium. (C) The production of EltA was analyzed in WT, Δ*arcA* mutant, Δ*arcA* complemented by western blotting with GroEL as an internal control. (D) The expression of *eltA* during ETEC adherence to IPEC-J2 cells were assessed by RT–qPCR. (E) Expression of *eltA* during infection in the mouse intestine was compared in WT, Δ*arcA* and Δ*arcA* complemented strains by RT–qPCR. Relative expression levels were calculated compared with the *tus* gene as an internal control, fold changes are relative to the WT. Data are presented as the mean ± standard deviation of triplicate samples from three independent experiments or from a pool of three mice. Significant differences were evaluated by one-way ANOVA, and the asterisks indicate **, P <

0.01 and *, $P < 0.05$. (F) An EMSA showing phosphorylated ArcA-His$_6$ binding to the *eltA* promoter region. This assay was performed according to Fig. 3F.

Fig. 5 Deletion of *arcA* significantly decreased *estB* expression. (A) Features of the *estB* promoter region. Italic and bold, start codon; bold, transcription start site; shaded, -35 and -10 regions; and underlined, predicted ArcA binding sites. (B and C) A lack of *arcA* led to downregulation of *eltA* in TSB media. Transcription of *estB-lacZ* (B) and production of STb (C) were examined in WT, Δ*arcA* mutant and Δ*arcA* complemented strains during growth in TSB media. Production of STb was analyzed by ELISA. (D) Expression levels of *estB* during ETEC adhering to IPEC-J2 cells were assessed by RT–qPCR. (E) Expression of *estB* during infection in the mouse intestine was compared in WT, Δ*arcA* and Δ*arcA* complemented strains by RT–qPCR. Relative expression levels were calculated compared with the *tus* gene as an internal control, fold changes are relative to the WT. Data are presented as the mean ± standard deviation of triplicate samples from three independent experiments or from a pool of three mice. Significant differences were evaluated by one-way ANOVA, and the asterisks indicate **, $P < 0.01$ and *, $P < 0.05$. (F) An EMSA showing phosphorylated ArcA-His$_6$ binding to the *estB* promotor region.

Fig. 6 ArcA positively regulated *stx2e* expression. (A) Features of the *stx2e* promoter region. Italic and bold, start codon; bold, transcription start site; shaded, -35 and -10 regions; and underlined, predicted ArcA binding sites. (B, C, D and E) A lack of *arcA* led to downregulation of *stx2e* in media, during ETEC-host cell interaction and *in vivo*. (B and C) A lack of *arcA* led to downregulation of *stx2e* in TSB media.

Transcription of *stx2e-lacZ* (B) and production of Stx2e (C) were examined in WT, Δ*arcA* mutant and Δ*arcA* complemented strains during growth in TSB media. The production of Stx2e was analyzed by western blotting with GroEL as an internal control. (D)The expression levels of *stx2e* during ETEC adherence to IPEC-J2 cells were assessed by RT–qPCR. (E) Expression of *stx2e* during infection in the mouse intestine was compared in WT, Δ*arcA* and Δ*arcA* complemented strains by RT–qPCR. Relative expression levels were calculated compared with the *tus* gene as an internal control, fold changes are relative to the WT. Data are presented as the mean ± standard deviation of triplicate samples from three independent experiments or from a pool of three mice. Significant differences were evaluated by one-way ANOVA, and the asterisks indicate **, $P < 0.01$. (F) An EMSA showing phosphorylated ArcA-His_6 binding to the *stx2e* promotor region.

Fig. 7 ArcA positively regulated *hlyCABD* expression. (A) Roles of *hlyA* and *hlyE* in hemolysis under microaerobic conditions, as revealed by a blood agar plate assay. (B) Features of the *hlyC* promoter region. Italic and bold, start codon; bold, transcription start site; shaded, -35 and -10 regions; and underlined, predicted ArcA binding sites. (C and D) A lack of *arcA* led to downregulation of *hlyA* in TSB media. Transcription of *hlyA-lacZ* (C) and production of HlyA (D) were examined in WT, Δ*arcA* mutant, Δ*arcA* complemented and Δ*hlyA* mutant strains during growth in TSB media. The production of HlyA was analyzed by western blotting with GroEL as an internal control. (E) Expression levels of *hlyA* during ETEC adhering to IPEC-J2 cells were assessed by RT–qPCR. (F) Expression of *hlyA* during infection in the mouse

intestine was compared in WT, Δ*arcA* and Δ*arcA* complemented strains by RT–qPCR. Relative expression levels were calculated compared with the *tus* gene as an internal control, fold changes are relative to the WT. Data are presented as the mean ± standard deviation of triplicate samples from three independent experiments or from a pool of three mice. Significant differences were evaluated by one-way ANOVA, and the asterisks indicate **, $P < 0.01$. (G) An EMSA showing phosphorylated ArcA-His_6 binding to the *hlyCABD* promotor region.

Fig. 8 Positive regulation of several virulence genes by ArcA requires H-NS. (A to C) Relative expression levels of *eltA* (A), *estB* (B), and *hlyC* (C) were assessed by RT–qPCR in WT, Δ*arcA*, Δ*hns* and Δ*arcA*Δ*hns* strains grown microaerobically at 37 °C in TSB medium. Relative expression levels were calculated compared with the *tus* gene as an internal control, and levels in the WT were set as 1.0. Data are presented as the mean ± standard deviation of triplicate samples from three independent experiments. Significant differences were evaluated by Student's *t* test, and the asterisks indicate **, $P < 0.01$ and *, $P < 0.05$. (D) An EMSA showing H-NS-FLAG binding to the *eltA* promotor region. (E) Competitive EMSA. Reactions containing 0.1 pmol *eltA* probe and 0.75 μM purified H-NS and either 0, 0.5, 1, 2, 3, 4 or 5 μM phosphorylated ArcA. Lanes labeled '–', 'H-NS', 'ArcA', and H-NS (–DNA) indicate no protein, 0.75 μM H-NS, 2 μM ArcA, and 0.75 μM H-NS without DNA, respectively. The reactions were run on polyacrylamide gels and stained with 1 × SYBR Gold nucleic acid-staining solution (top panel) and then transferred to nitrocellulose membrane and probed for H-NS (middle panel) and ArcA (bottom

panel) using anti-FLAG and for anti-His_6 antibodies, respectively.

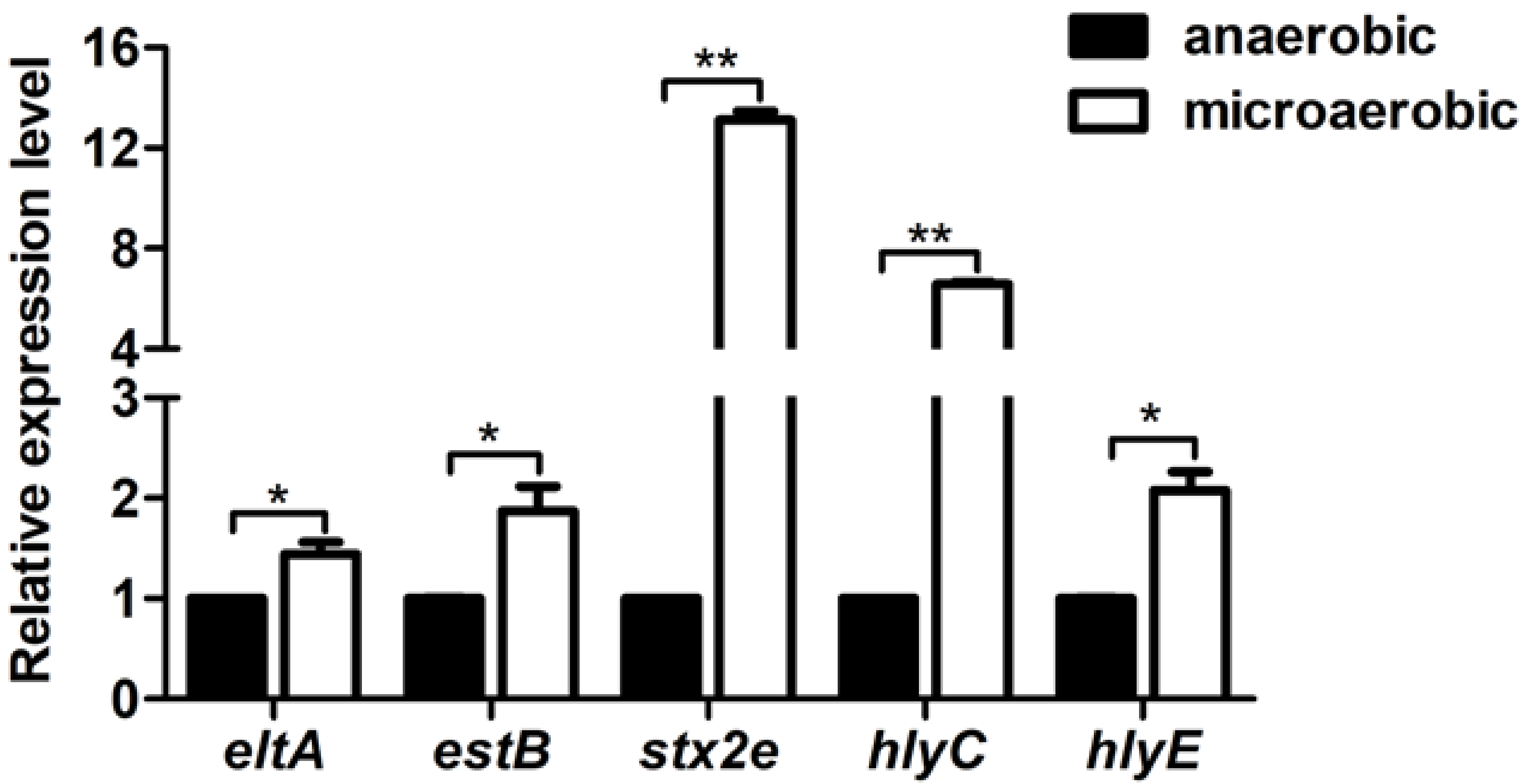

anaerobic
microaerobic
Relative expression level
16
12
8
4
3
2
1
0
**
**
*
*
*
eltA
estB
stx2e
hlyC
hlyE

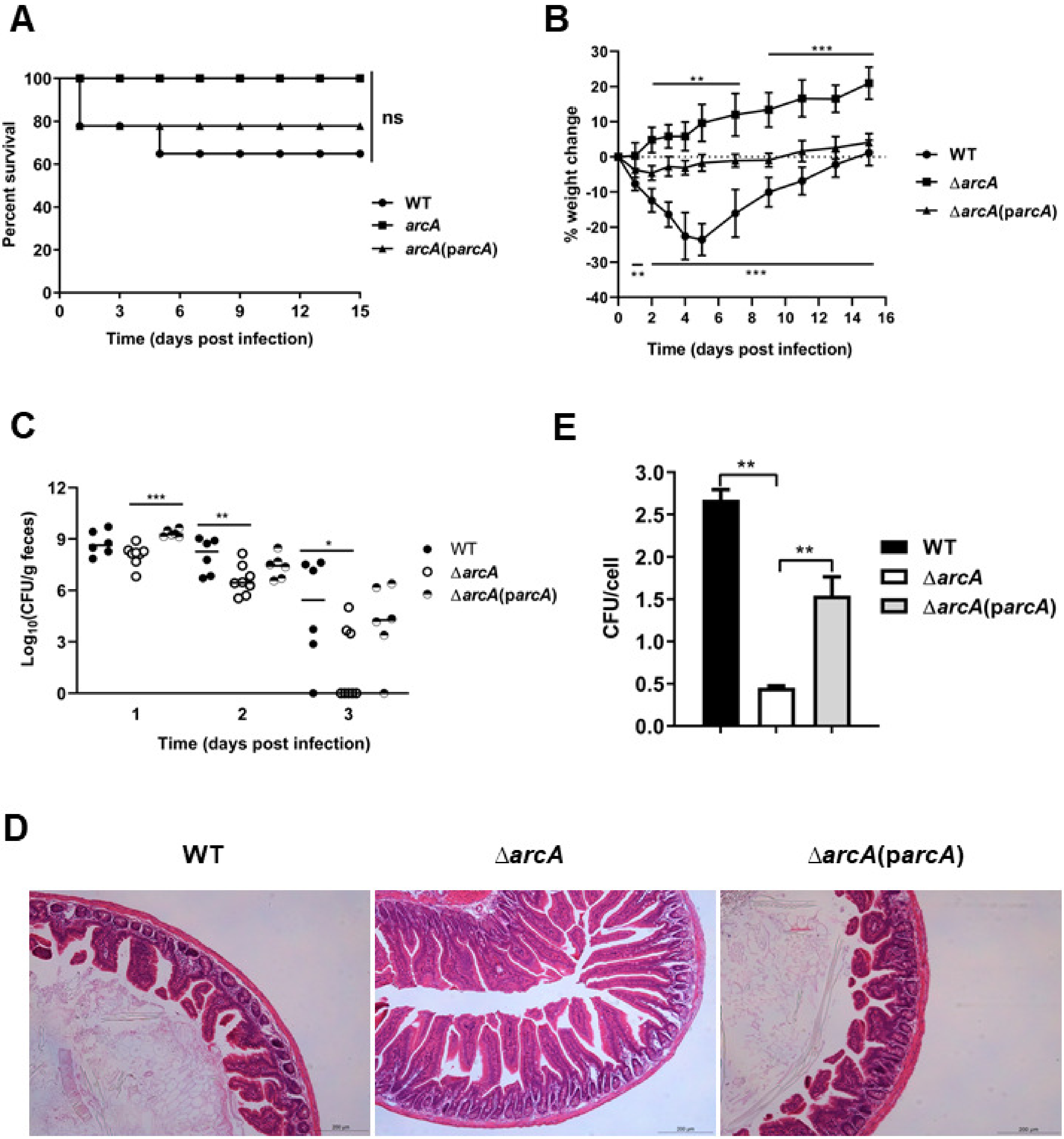
A
Percent survival
Time (days post infection)
ns
WT
arcA
arcA(parcA)
B
% weight change
Time (days post infection)
WT
ΔarcA
ΔarcA(parcA)
C
Log10(CFU/g feces)
Time (days post infection)
WT
ΔarcA
ΔarcA(parcA)
E
CFU/cell
WT
ΔarcA
ΔarcA(parcA)
D
WT
ΔarcA
ΔarcA(parcA)

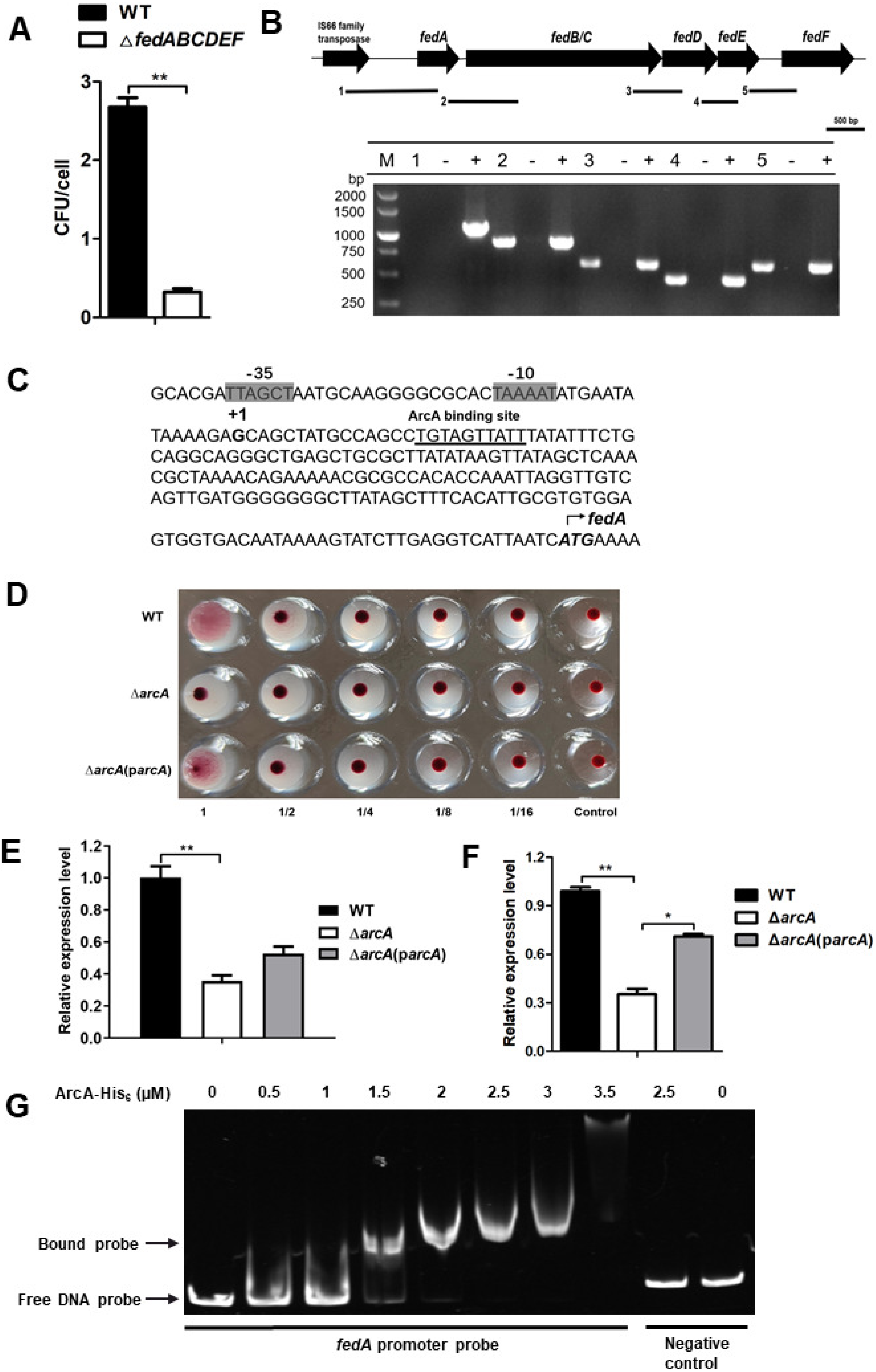
A
WT
△fedABCDEF
**
CFU/cell
3
2
1
0
B
IS66 family transposase
fedA
fedB/C
fedD
fedE
fedF
1
2
3
4
5
500 bp
M 1 - + 2 - + 3 - + 4 - + 5 - +
bp
2000
1500
1000
750
500
250
C
-35
-10
GCACGATTAGCTAATGCAAGGGGCGCACTAAAATATGAATA
+1
ArcA binding site
TAAAAGAGCAGCTATGCCAGCCTGTAGTTATTTATATTTCTG
CAGGCAGGGCTGAGCTGCGCTTATATAAGTTATAGCTCAAA
CGCTAAAACAGAAAAACGCGCCACACCAAATTAGGTTGTC
AGTTGATGGGGGGGCTTATAGCTTTCACATTGCGTGTGGA
fedA
GTGGTGACAATAAAAGTATCTTGAGGTCATTAATCATGAAAA
D
WT
ΔarcA
ΔarcA(parcA)
1
1/2
1/4
1/8
1/16
Control
E
Relative expression level
1.2
1.0
0.8
0.6
0.4
0.2
0.0
**
WT
ΔarcA
ΔarcA(parcA)
F
Relative expression level
1.2
0.9
0.6
0.3
0.0
**
*
WT
ΔarcA
ΔarcA(parcA)
G
ArcA-His6 (μM)
0
0.5
1
1.5
2
2.5
3
3.5
2.5
0
Bound probe
Free DNA probe
fedA promoter probe
Negative control

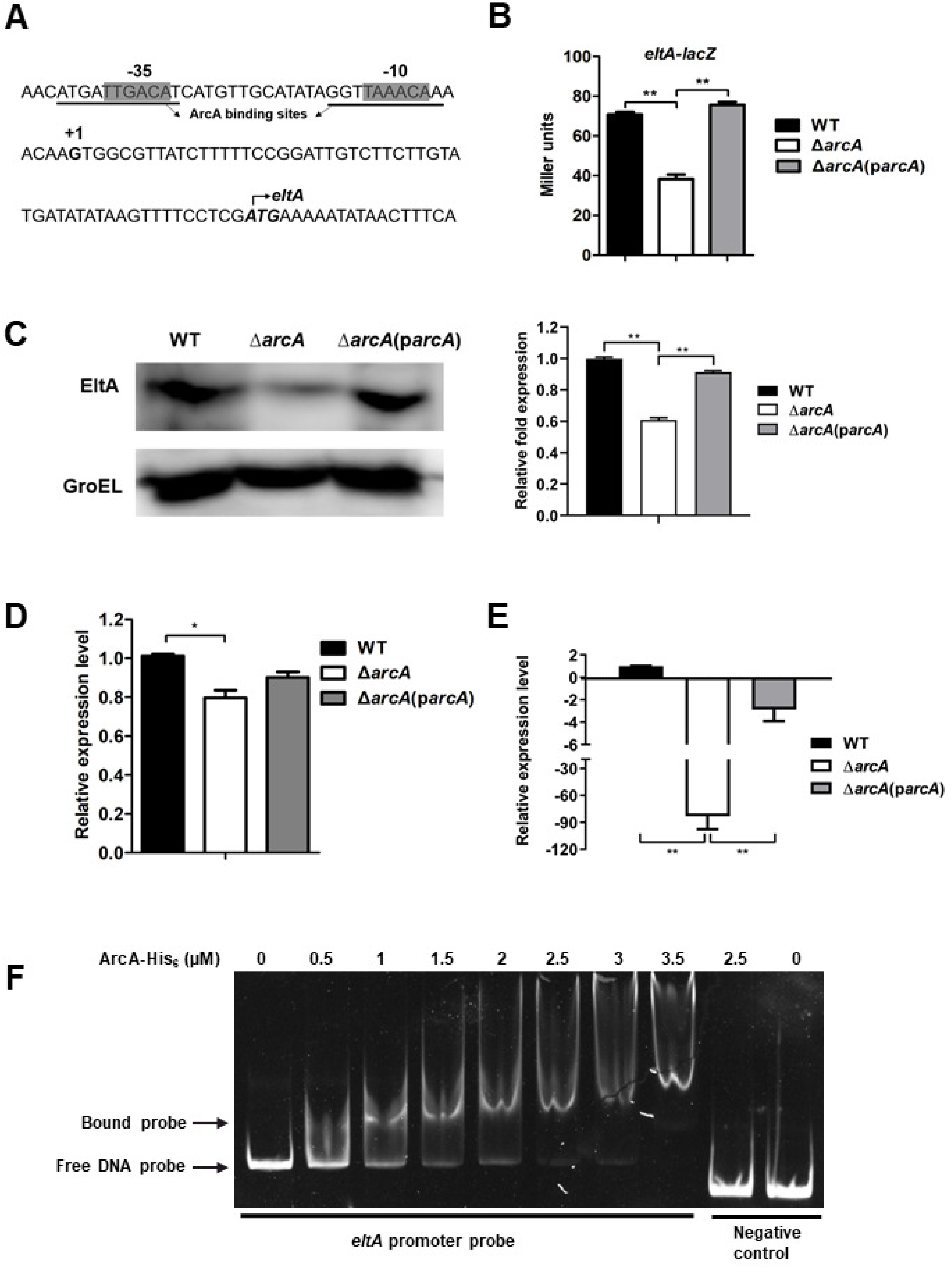

A
-35
-10
AACATGATTGACATCATGTTGCATATAGGTTAAACAAA
ArcA binding sites
+1
ACAAGTGGCGTTATCTTTTTCCGGATTGTCTTCTTGTA
eltA
TGATATATAAGTTTTCCTCGATGAAAAATATAACTTTCA
B
eltA-lacZ
Miller units
WT
ΔarcA
ΔarcA(parcA)
C
WT
ΔarcA
ΔarcA(parcA)
EltA
GroEL
Relative fold expression
D
Relative expression level
E
Relative expression level
F
ArcA-His6 (μM)
0 0.5 1 1.5 2 2.5 3 3.5 2.5 0
Bound probe
Free DNA probe
eltA promoter probe
Negative control

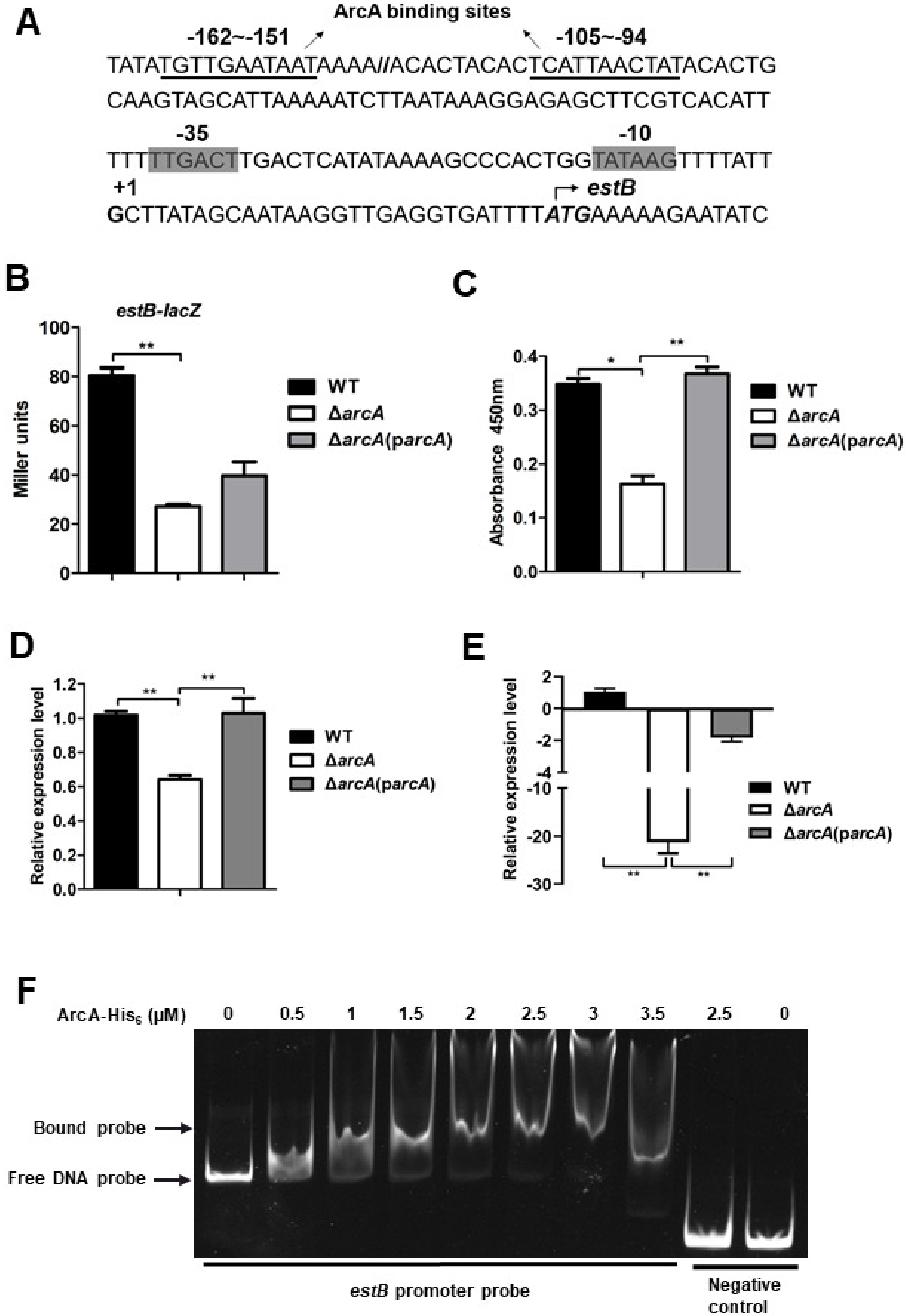
A
ArcA binding sites
-162~-151
-105~-94
TATATGTTGAATAATAAAA//ACACTACACTCATTAACTATACACTG
CAAGTAGCATTAAAAATCTTAATAAAGGAGAGCTTCGTCACATT
-35
-10
TTTTTGACTTGACTCATATAAAAGCCCACTGGTATAAGTTTTATT
+1
estB
GCTTATAGCAATAAGGTTGAGGTGATTTTATGAAAAAGAATATC
B
estB-lacZ
Miller units
WT
ΔarcA
ΔarcA(parcA)
C
Absorbance 450nm
D
Relative expression level
E
F
ArcA-His6 (μM)
0 0.5 1 1.5 2 2.5 3 3.5 2.5 0
Bound probe
Free DNA probe
estB promoter probe
Negative control

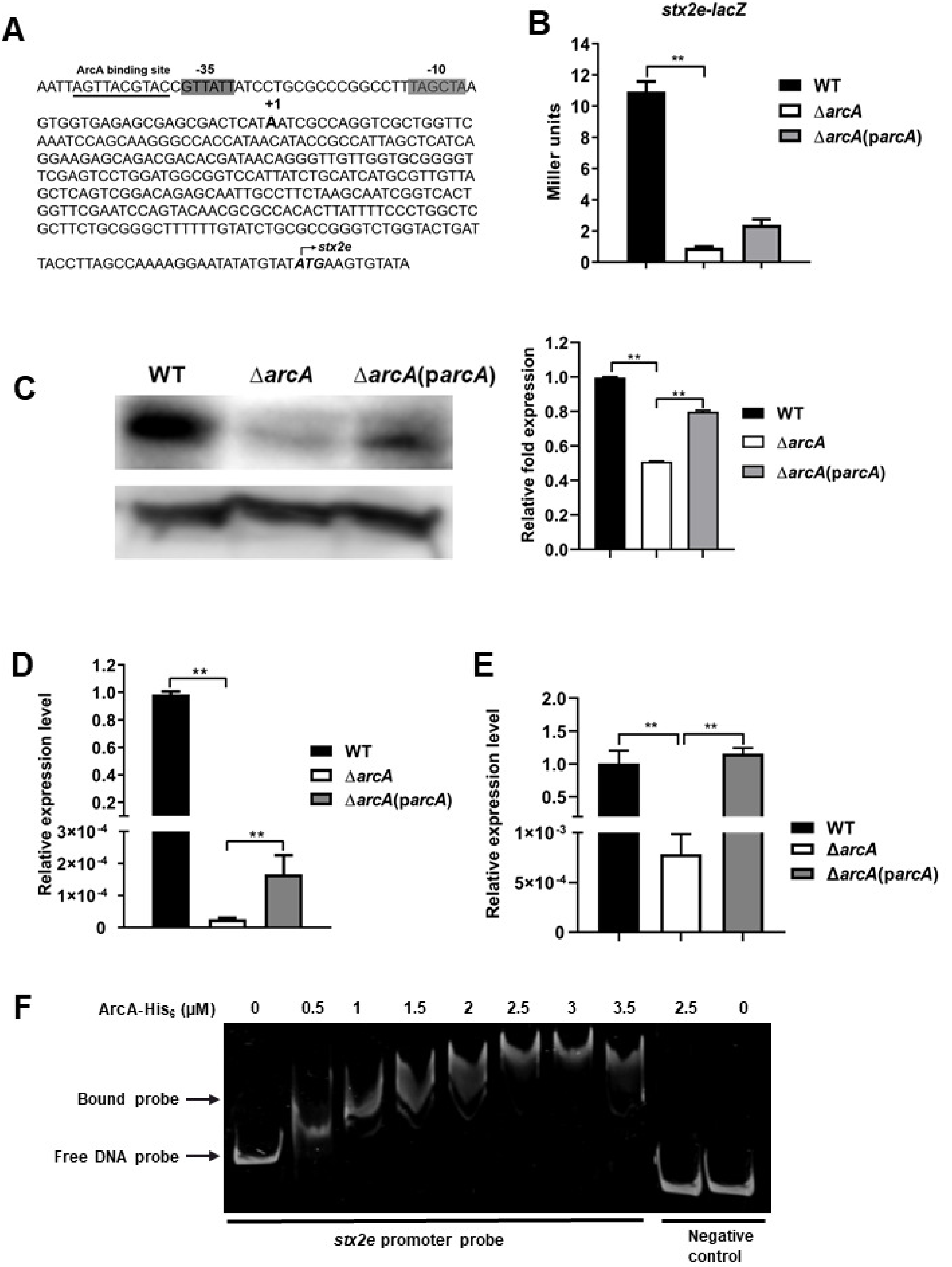

A
ArcA binding site
-35
-10
AATTAGTTACGTACCGTTATTATCCTGCGCCCGGCCTTTAGCTAA
+1
GTGGTGAGAGCGAGCGACTCATAATCGCCAGGTCGCTGGTTC
AAATCCAGCAAGGGCCACCATAACATACCGCCATTAGCTCATCA
GGAAGAGCAGACGACACGATAACAGGGTTGTTGGTGCGGGGT
TCGAGTCCTGGATGGCGGTCCATTATCTGCATCATGCGTTGTTA
GCTCAGTCGGACAGAGCAATTGCCTTCTAAGCAATCGGTCACT
GGTTCGAATCCAGTACAACGCGCCACACTTATTTTCCCTGGCTC
GCTTCTGCGGGCTTTTTTGTATCTGCGCCGGGTCTGGTACTGAT
stx2e
TACCTTAGCCAAAAGGAATATATGTATATGAAGTGTATA
B
stx2e-lacZ
Miller units
**
WT
ΔarcA
ΔarcA(parcA)
C
WT
ΔarcA
ΔarcA(parcA)
Relative fold expression
**
**
WT
ΔarcA
ΔarcA(parcA)
D
Relative expression level
**
**
WT
ΔarcA
ΔarcA(parcA)
E
Relative expression level
**
**
WT
ΔarcA
ΔarcA(parcA)
F
ArcA-His6 (μM)
0
0.5
1
1.5
2
2.5
3
3.5
2.5
0
Bound probe
Free DNA probe
stx2e promoter probe
Negative control

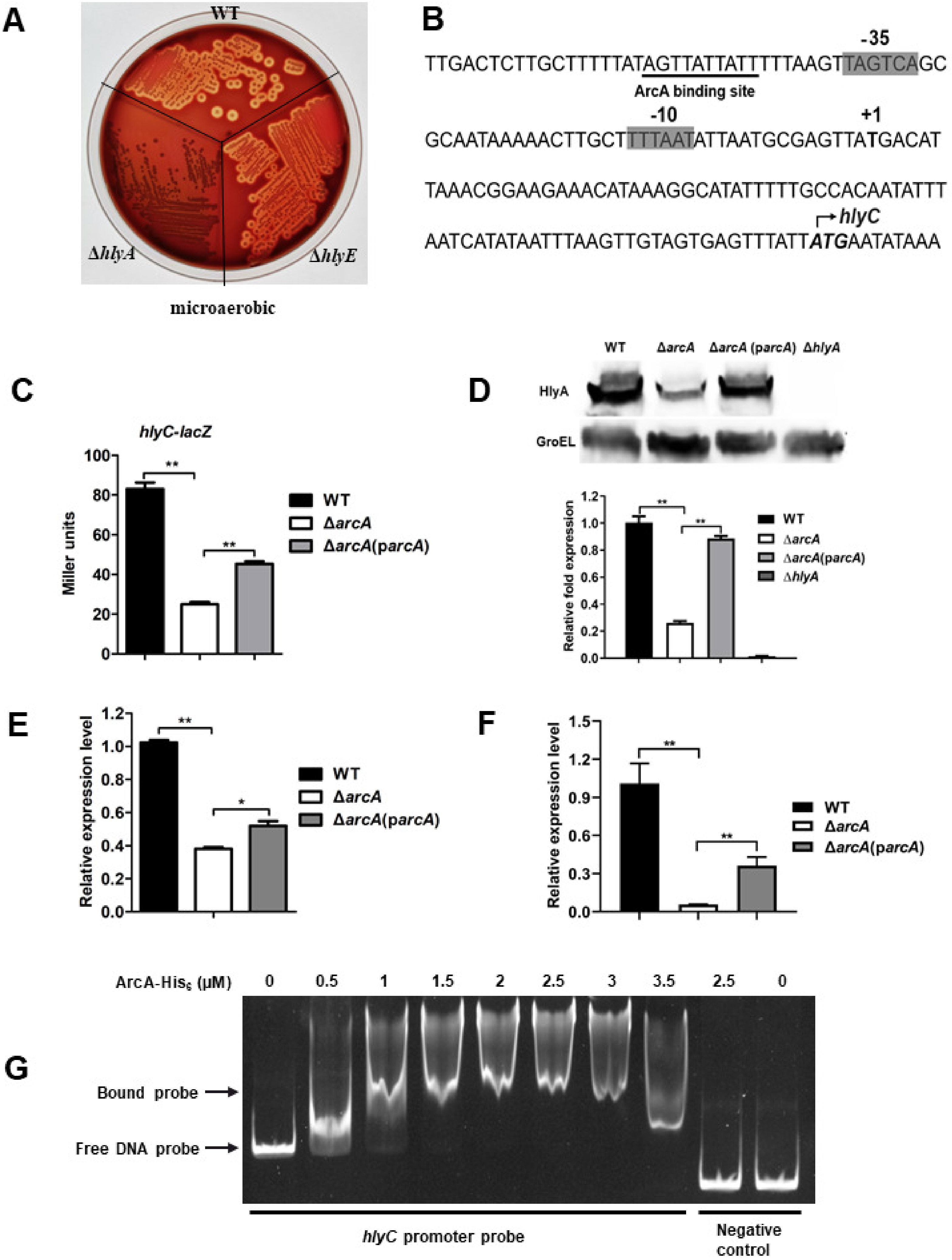
A
WT
ΔhlyA
ΔhlyE
microaerobic
B
-35
TTGACTCTTGCTTTTTATAGTTATTATTTTTAAGTTAGTCAGC
ArcA binding site
-10
+1
GCAATAAAAACTTGCTTTTAATATTAATGCGAGTTATGACAT
TAAACGGAAGAAACATAAAGGCATATTTTTGCCACAATATTT
hlyC
AATCATATAATTTAAGTTGTAGTGAGTTTATTATGAATATAAA
C
hlyC-lacZ
Miller units
WT
ΔarcA
ΔarcA(parcA)
D
WT
ΔarcA
ΔarcA (parcA)
ΔhlyA
HlyA
GroEL
Relative fold expression
WT
ΔarcA
ΔarcA(parcA)
ΔhlyA
E
Relative expression level
WT
ΔarcA
ΔarcA(parcA)
F
Relative expression level
WT
ΔarcA
ΔarcA(parcA)
G
ArcA-His6 (μM)
0
0.5
1
1.5
2
2.5
3
3.5
2.5
0
Bound probe
Free DNA probe
hlyC promoter probe
Negative control

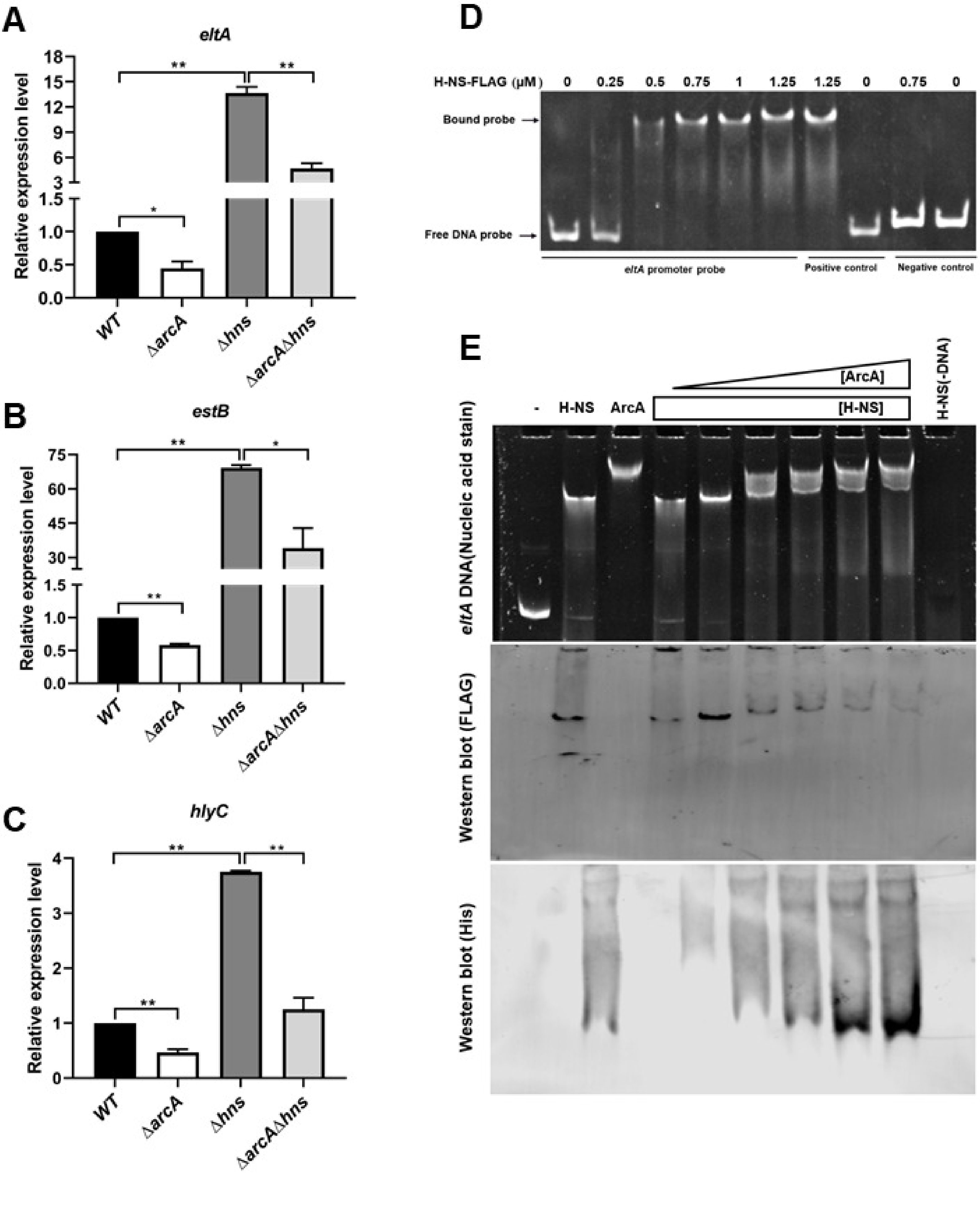

A
eltA
Relative expression level
WT
ΔarcA
Δhns
ΔarcAΔhns
B
estB
C
hlyC
D
H-NS-FLAG (μM)
0
0.25
0.5
0.75
1
1.25
1.25
0
0.75
0
Bound probe
Free DNA probe
eltA promoter probe
Positive control
Negative control
E
[ArcA]
-
H-NS
ArcA
[H-NS]
H-NS(-DNA)
eltA DNA(Nucleic acid stain)
Western blot (FLAG)
Western blot (His)
